# Selected Topics in Time Series Forecasting: Statistical Models vs. Machine Learning

**DOI:** 10.3390/e27030279

**Published:** 2025-03-07

**Authors:** Dag Tjøstheim

**Affiliations:** Department of Mathematics, University of Bergen, 5008 Bergen, Norway; dag.tjostheim@uib.no

**Keywords:** time series forecasting, machine learning, statistical model, neural network, random forest, forecasting competition, weather forecasting

## Abstract

Machine learning forecasting methods are compared to more traditional parametric statistical models. This comparison is carried out regarding a number of different situations and settings. A survey of the most used parametric models is given. Machine learning methods, such as convolutional networks, TCNs, LSTM, transformers, random forest, and gradient boosting, are briefly presented. The practical performance of the various methods is analyzed by discussing the results of the Makridakis forecasting competitions (M1–M6). I also look at probability forecasting via GARCH-type modeling for integer time series and continuous models. Furthermore, I briefly comment on entropy as a volatility measure. Cointegration and panels are mentioned. The paper ends with a section on weather forecasting and the potential of machine learning methods in such a context, including the very recent GraphCast and GenCast forecasts.

## 1. Introductory Remarks

To be able to succeed in time series forecasting, a repeated past pattern is needed, and that pattern has to continue into the future to some extent. The higher the regularity of this pattern, the higher its expected forecastability. In other words, the higher the disorder is in the history of the time series, the more difficult it is to predict. The disorder of a time series can be formally measured in terms of its entropy (approximate entropy and sample entropy), e.g., see Ref. [1]. The concept of entropy comes from the field of physics. In information theory, the Shannon interpretation of entropy is used to measure the amounts of information required to accurately send and receive some messages (e.g., see the explanation and gedanken experiment in [2]). In data analysis, the concept of entropy has been used to analyze patterns in medical data, such as heart rate; later, this spread to applications in physiology, finance, climate sciences, and a number of other phenomena. In practice, the entropy in a forecasting situation is seldomly computed explicitly, but it is indirectly reflected in the uncertainty of a time series forecast. Uncertainty can be given in terms of a probability interval, a confidence interval, or, in more detail, as a forecast distribution, which attaches probabilities and distributions to a variety of predicted values. Even though a forecast is often just given by a point forecast, which may be interpreted as the most likely value of a time series variable at a future time, it seems to be increasingly common nowadays to let the point forecast be accompanied by a measure of uncertainty. This, in a sense, can be thought of as reflecting the entropy of the series. It is explained in more detail at the end of Section 5.2. A few more comments on entropy and forecastability are also given in Section 4.4 on forecasting competitions.

The history of time series forecasting goes very far back, at least to the age of antiquity, with more or less accurate predictions of astronomic events and future weather. Presently, forecasts are used virtually everywhere, ranging from, say, economics and opinion polls to long- and short-term weather forecasts. Some forecasts are more or less purely statistical in nature, i.e., demand forecasting for groups of items based entirely and exclusively on past sales, but other types of forecasts, such as financial predictions, are aided by economic theory, whereas weather forecasting may depend critically on a geophysical theory of the atmosphere.

At present, forecasting is helped by an enormous increase in the availability of data. Ours is truly the age of Big Data. However, how can this help us to make better predictions? Is it easier or more difficult to see patterns in the data that can be exploited in forecasting? In parallel with the increase in data, there has been an increase in the availability and speed of computers. Computers can certainly detect patterns in complex data that humans or more simple analyzing techniques cannot detect. There are machine learning (hereafter denoted ML) techniques for handling such patterns, but are they useful for forecasting? Or does the computer see patterns in the noise and, in that way, make misleading forecasts?

I will treat these questions in this paper. It is a review where I seek to include very recent contributions. I emphasize attempts to find an answer and to pinpoint situations where ML does particularly well or where it does not work so well. A frequent criticism of machine learning is that it is a black box method. Nevertheless, can something be said when it comes to finding reasons for it doing well in some situations, e.g., beating simple parametric models, and not so well in other situations, where it is beaten by other types of models? See the discussion in Section 4.3 and at the end of Section 6.

What is the potential for ML methods in the future? Are these methods improving in quality while parametric models are at a standstill? Moreover, how well can purely algorithmic ML methods be integrated into models to a large degree based on physics and non-statistical features, such as in weather forecasting?

Partially, I try to give my review a somewhat personal touch. It is colored by my research background, which has not been focused directly on forecasting but has been centered on topics close to the problem of forecasting. Hence, I will discuss nonstationarity and the patterns of nonstationarity that are statistically forecastable, such as cointegration. This has also led to looking at leading lags and Granger causality, as outlined in [3]. In addition, I will mention the possible potential of local Gaussian models presented in [4,5]. Further, I briefly look at GARCH-like structures, not only in volatility prediction for financial series, but in integer time series modeling, so-called Poisson autoregression, and similar recursive schemes for continuous time data described by stochastic differential equations leading to the connection with Hawkes processes. There seem to be only limited applications of ML methods in these contexts.

To compare individual forecasting methods and types of methods is a challenging task. What kind of criteria or metrics should be used? A number of forecasting competitions have been arranged, and I report on some of the main ones in Section 4.4. In most of these competitions, forecasting accuracy has been used as a yardstick, but then several measures of accuracy exist, and the ranking of methods may depend on the measure used. However, clearly, there are other key metrics in judging forecasting algorithms, such as training time, interpretability, and robustness against noisy data. I will briefly discuss each of these criteria for the methods treated in the present paper.

Here is an outline of the paper: In Section 2, I mention the Holt-Winters models, which, in many ways, may be said to have started modern applied forecasting. These models are intuitive, provide good results, and are still used. A related, so-called “θ-model”, has been doing well in forecasting competitions. Actually, this model was declared the winner of the M3 forecasting competition; see [6]. The well-known ARIMA models, also briefly discussed in Section 2, are related to the Holt-Winters models. Both point forecasts and interval forecasts can be provided in the Gaussian case in particular.

All of these models are linear (and quite often Gaussian). Section 3 is devoted to simple nonlinear parametric models and to nonparametric forecasting.

Section 4 is, in some sense, the main part of the paper. Here, I discuss the various types of ML forecasts, such as those based on neural networks and random forest, which are possibly the two main contenders for simpler statistical models. What are their strengths and weaknesses? How and when can they be strengthened further by combining various ML forecasts with classic statistical models? How well do they do in forecasting competitions (covered in Section 4.4) compared to more traditional methods? Further, parametric models can be analyzed theoretically. How about ML methods?

Section 5 continues the discussion of ML methods. I treat multivariate time series and panels, nonstationary effects, and cointegration as an alternative instrument to differencing and removing deterministic trends. Further, I discuss forecasting distributions, including scores, forecasting count time series via GARCH-type structures, and analogs in continuous time with potential applications in multivariate financial forecasting and contagion. For some of these problems, ML methods seem less well developed than in forecasting stationary one-dimensional series.

Finally, in Section 6, I look at weather forecasting. Recently, ML methods have been introduced at various stages of the forecasting process, sometimes eminently successful but at other times, a failure. What is the future of ML forecasting here? This is discussed in light of the very recent ML methods of GraphCast and GenCast.

I do not give any explicit forecasting examples using my own real data. For a great number of such examples, I refer, instead, to the discussion and references in Section 4.4.

## 2. Classical Parametric Forecasts

The earliest forecasting technique that is still being used is probably the exponential smoothing technique based on work in [7,8]. Exponential smoothing is an example of a recursive scheme, with such schemes being extensively used in increasingly complex forms in modern nonlinear and ML forecasting. The basic idea goes back to at least Poisson as an extension of a numerical analysis technique from the 17th century, and it was later adopted by the signal-filtering processing community in the 1940s.

Let $\left\{y_{t}\right\}$ be a time series for which predictions ${\hat{y}}_{t+1}$ at time t+1 are sought. Then, the simplest form of an exponential smoothing forecast is given by

(1) $${\hat{y}}_{t+1}=\alpha y_{t}+\left(1-\alpha \right){\hat{y}}_{t}.$$

By solving recursively, ${\hat{y}}_{t+1}$ is expressed as an exponentially weighted average of the previous observations of $\left\{y_{t}\right\}$, from which the appellation exponential smoothing is derived. The parameter α(0≤α≤1) must be estimated, usually using least squares, and the initial value ${\hat{y}}_{0}$ must be stipulated by, for example, taking an average of the early values of $\left\{y_{t}\right\}$, which are needed for starting the recursive scheme.

Simple exponential smoothing does not work well if there is a trend involved. Including an (instantaneous) trend estimate, $\left\{{\hat{d}}_{t}\right\}$, results in a double exponential smoothing scheme:

(2) $${\hat{y}}_{t+1}=\alpha y_{t}+\left(1-\alpha \right){\hat{y}}_{t}+{\hat{d}}_{t}$$

(3) $${\hat{d}}_{t+1}=\beta \left({\hat{y}}_{t+1}-{\hat{y}}_{t}\right)+\left(1-\beta \right){\hat{d}}_{t}$$

where α is the data smoothing factor, and β(0≤β≤1) is the trend smoothing factor. These factors and the initial conditions can be determined as indicated above for the simple scheme in (1). The inclusion of a trend also makes it easier to forecast several steps ahead.

With the help of a student (Peter Winters), Holt extended the scheme further to contain seasonal indices, the Holt-Winters forecasts, involving triple exponential smoothing. See also [9]. Exponential smoothing can be extended to multivariate time series; see [10].

Probably the most well-known class of parametric time series is the class of ARIMA (integrated autoregressive moving average) models. The major breakthrough of this class of models came in the form of a book [11]. The authors introduced, in a time series context, the classical sequence of statistical modeling: identification, estimation, and diagnostic checking. The ARIMA models are so well-known that it seems unnecessary to say much about them here, but a few facts will be mentioned for completeness. The simplest member of this class is the purely recursive autoregressive model (the models are assumed to have a zero mean),

(4) $$y_{t}=a_{1}y_{t-1}+\cdots +a_{p}y_{t-p}+e_{t},$$

where $a_{1},\ldots ,a_{p}$ are unknown parameters in a *p*th-order model, and $\left\{e_{t}\right\}$ is a series of uncorrelated (in inference, these are often assumed to be iid (independent identically distributed)) variables. The process is stationary if the roots of the characteristic polynomial $A\left(z\right)=1-a_{1}z-\cdots -a_{p}z^{p}$ are located outside the unit circle in the *z*-plane. The one-step-ahead forecast is given by ${\hat{y}}_{t+1}=a_{1}y_{t}+\cdots +a_{p}y_{t-p+1}$, and higher-order forecasts are given by reiterating this equation. The estimation of the parameters is carried out using maximum likelihood or quasi-maximum likelihood, and the asymptotic confidence intervals and asymptotic distribution of the forecasts are easily established under weak regularity conditions. The order is determined by inspecting the so-called partial autocorrelation function or by using an AIC-type criterion.

The class of ARMA(p,q) models is formed by replacing the noise or innovation term $e_{t}$ with a moving average term, resulting in

(5) $$y_{t}-a_{1}y_{t-1}-\cdots -a_{p}y_{t-p}=e_{t}+b_{1}e_{t-1}+\cdots +b_{q}e_{t-q}.$$

It is somewhat more cumbersome to obtain parameter estimates and one-step or multi-step forecasts using maximum likelihood or quasi-maximum likelihood, but standard computer packages are available, where forecast confidence intervals are computed.

ARMA models can be further extended to encompass nonstationary models with a linear stochastic or deterministic trend. This is carried out by reducing the series to a stationary series by differencing the observations up to *d* times, resulting in an ARIMA(p,d,q) model. Often, d=1 is sufficient. The models can be extended by including a seasonal component that, in itself, can be modeled by an ARIMA-type process. In the vector time series case, the ARIMA is based on the individual differencing of the component series, but presently, this structure is predominantly replaced by a cointegration approach; this will be briefly mentioned at the end of Section 5.3.

Forecasts can be computed using uncertainty intervals from standard statistical packages. The ARIMA models have tended to do well in forecasting competitions. They have been compared to Holt-Winters exponential models, but it is not correct that these models are merely a subset of ARIMA models; this is because Holt-Winters models contain instantaneous estimates of trends and seasonal components. These quantities are recursively updated in time, which is not the case for ARIMA models.

The ARIMA models are easily updated to multivariate series $\left\{y_{t}\right\}$ by replacing the scalar parameters $a_{1},\ldots ,a_{p}$ and $b_{1},\ldots ,b_{q}$ with matrices. The identification, estimation, and validation steps are more complex. A useful reference is [12]. There are several program packages for more or less automatically processing multivariate ARIMA models (see, e.g., a Python 3.13 package on forecasting for ARIMA models and more general machine learning forecasts regarding multivariate time series). These models can also be allowed to contain a possible moving average exogenous variable, $x_{t}$, in addition to the multivariate innovation term $e_{t}$. These are the so-called ARIMAX models. Again, forecasts with uncertainty intervals can be obtained from program packages.

The winner of the Makridakis M3 [6] forecasting competition was a forecast obtained using the so-called θ (theta) method. The method was introduced in [13]. It is much more completely described in the monograph [14]. It can be seen as a generalization of Holt-Winters-type models. The choice of weight parameters and the numbers of trend functions (and their nature) has been researched extensively; this is reflected in, e.g., Ref. [15], in a comparison with machine learning forecasts.

In the general literature on theta models, there are two strands. The first one gives probabilistic properties of the method, starting in [16]; subsequently, a number of theoretical results was collected in [14]. The latter provides a complete analysis of the theta method under a unit root-generating process, explaining its success in the M3 Makridakis competition in 2000 [6]. The authors of Ref. [14] also introduced the multivariate theta method and looked at its relationship to cointegration.

The second strand expands and details various implementations of the method, optimizing theta models and pointing out their relationship with state space models (see [17]).

A recent generalization of the theta method is given in [18]. Connections to adaptive learning forecasts were pursued in [19].

The connection of the theta method to the perhaps more well-known class of state space models prompts a short mention of the structure of the latter. In fact, state space models are very powerful and versatile tools for working with time series and their forecasting. They were initially developed by engineers but have been widely adopted and extended among other applications to econometrics. Two central textbooks are [20,21]. State space models are formulated by means of two different equations: an equation in terms of the states, $\left\{\alpha_{t}\right\}$, which are a set of variables that are usually unobserved. Typical examples are trends, seasonal components, or time-varying parameters. The state equation describes the dynamic law governing the states for two adjacent points in time. The second is the observation equation, which specifies the relationship between observed data and unobserved states. A linear Gaussian version of such a system could look like this:

(6) $$\alpha_{t+1}=T_{t}\alpha_{t}+\Gamma_{t}+R_{t}\eta_{t},\;\eta_{t}∼N\left(0,Q_{t}\right)$$

(7) $$y_{t}=E_{t}\alpha_{t}+D_{t}+C_{t}\varepsilon_{t}\;\varepsilon_{t}∼N\left(0,H_{t}\right)$$

with the initial condition $\alpha_{1}∼N\left(\alpha_{0},P_{0}\right)$. In these equations, $\eta_{t}$ and $\varepsilon_{t}$ are the state and observational vectors of zero-mean Gaussian noises. The matrices $T_{t},\Gamma_{t},R_{t},Q_{t},E_{t},D_{t},C_{t}$, and $H_{t}$ are the so-called (generally time-varying) system matrices, and $\alpha_{1}$ and $P_{1}$ are the initial state and state covariance matrix, respectively.

Once such a system is fully specified, the core problem is to provide optimal predictions of states and their covariances over time. This can be carried out by looking back in time using the well-known Kalman filter.

Given any set of data and a specific model, the system is not fully specified in most cases because this would depend on unknown parameter matrices. An estimation of such parameters is sought, carried out using maximum likelihood defined by prediction error decomposition [20].

A more recent text book is [22]. The forecasting aspect is covered in, e.g., Ref. [23].

Concerning the key metrics mentioned in the Introduction, the classical parametric forecasting methods reported in the present section must be said to be easy to interpret, possibly with the exception of the theta-type models; see [15,24] for a simplification and rephrasing of the model in terms of more understandable terms. The training of the parametric models consists of estimating the parameters. This is very simply carried out for univariate models, whereas high-dimensional ARIMA models and state space models would require more effort. Usually, parametric models would contain a safeguard against overfitting by using an AIC-type criterion or a penalty factor, thus limiting the number of allowed parameters. Classical parametric models have high accuracy for forecasting in linear models, but it is not difficult to find nonlinear models where they fail completely.

## 3. Nonlinear and Nonparametric Forecasts

Up to now, I have been mostly concerned with linear and often Gaussian models. A claim of the ML methods is that (at least sometimes) they are capable of handling nonlinearities better than classical parametric models. This means that, in a sense, ML methods try to capture more of the probabilistic structure of the data. In comparison, ARIMA models are based solely on statistical moments, e.g., the covariance structure. This is fine if the data are Gaussian, but in the non-Gaussian case, one would expect that information may have been lost.

Before I go over to the main theme of this paper—the construction of ML methods, including their claimed ability to handle nonlinearities in a better way—it is instructive to take a look at classical parametric nonlinear models, including a look at the classical nonparametric methodology.

To make things simple, I concentrate on the first-order scalar model, where only one lag is included. Going from model to forecast is then trivial.

The most popular nonlinear model is probably the threshold model introduced in [25]. In its simplest form, it is given by

(8) $$y_{t}=\theta_{1}y_{t-1}\left\{s_{t-1}\leq c\right\}+\theta_{2}y_{t-1}\left\{s_{t-1}>c\right\}+e_{t}.$$

The process moves as a mixture of two autoregressive regimes, with autoregressive coefficients $\theta_{1}$ and $\theta_{2}$, respectively. The regime is determined by the state process, $\left\{s_{t}\right\}$, and a threshold, *c*. When $s_{t-1}\leq c$, it moves according to $\theta_{1}$ when $s_{t-1}>c$ according to $\theta_{2}$. The state process, $s_{t}$, may be equal to $y_{t-d}$ for a certain delay, *d*. An inference theory for the threshold process is given in [26].

There are numerous generalizations and applications of the threshold model; see, e.g., Refs. [27,28]. Usually, a threshold process is assumed to be stationary. For a nonstationary cointegration-type threshold process, see [29]. For a recent paper that covers the use of thresholds in neural networks, see [30].

The threshold model is sometimes criticized, perhaps especially in econometrics, for its abrupt change between regimes. There is a class of models with continuous transition between the $\theta_{1}$ and $\theta_{2}$ regimes, the so-called STAR models (smooth transition models). In the LSTAR model, the transition is modeled by a logistic function,

(9) $$y_{t}=\left(\theta_{1}+\theta_{2}G\left(\gamma ,c,s_{t-1}\right)\right)y_{t-1}+e_{t}$$

with *G* having the logistic form

(10) $$G\left(\gamma ,c,s_{t-1}\right)={\left(1+\exp\left\{-\gamma \left(s_{t-1}-c\right)\right\}\right)}^{-1},\;\gamma >0.$$

Alternatively, an exponential form, ESTAR, can be used:

(11) $$G\left(\gamma ,c,s_{t-1}\right)=1-\exp\left\{-\gamma {\left(s_{t-1}-c\right)}^{2}\right\}\;\gamma >0.$$

An earlier version of these models was introduced in [31]. Later, the author of Ref. [32] defined a family of STAR models that included both the LSTAR and ESTAR models and devised a data-driven strategy with the aim of, among other things, helping the user choose between these two alternatives. A number of generalizations of the simple models (10) and (11) exist. An update can be found in [33].

The last class of parametric nonlinear models is the hidden Markov chain autoregressive process. In contrast, for the threshold models and the STAR models, the state process is typically given by $s_{t}=y_{t-d}$, a delay of the observed process, in the hidden Markov chain autoregressive process. $\left\{s_{t}\right\}$ is a Markov chain so that

$$y_{t}=s_{t-1}y_{t-1}+e_{t},$$

where, in the simplest case, $s_{t}$, can only take two values, $\theta_{1}$ and $\theta_{2}$, say, and where $s_{t}$ varies between these two values according to a 2 × 2 Markov transition matrix. Both $\theta_{1}$ and $\theta_{2}$ and the entries in the probability transition matrix are unknown and have to be estimated. The EM algorithm is often used for this task [34]. Additionally, the mean and variance of the series could be the subject of Markov regime shifts [35]; see the more recent textbook reference also [36].

For all of these classes of processes, it is possible to do asymptotic inference on the parameters, and asymptotic confidence intervals can be secured under certain regularity conditions. In principle, these can be transferred to asymptotic uncertainty intervals for the forecasts. However, it seems more difficult to produce a distributional theory of forecasts, unlike the nonparametric models examined next.

The well-known kernel density estimate for the marginal density, p(y), of a stationary time series, $\left\{y_{t}\right\}$, given *n* observations $y_{1},\ldots ,y_{n}$, is given by

$$\hat{p}\left(y\right)=\sum_{t=1}^{n}K_{h}\left(y_{t}-y\right),$$

where K(·) is a kernel function, *h* is the bandwidth, and $K_{h}\left(\cdot \right)=h^{-1}K\left(h^{-1}\left(\cdot \right)\right)$. Under nonrestrictive conditions, consistency and asymptotic normality can be proved. It is well-known that the optimal (with respect to least squares deviation) predictor ${\hat{y}}_{t+1}$ is given by the conditional mean of $y_{t+1}$, given the past value. The conditional mean $m(y_{t+1}|y)$ of $y_{t+1}$ given $y_{t}=y$ can be estimated using the Nadaraya-Watson estimator

$$\hat{m}\left(y_{t+1}|y_{t}=y\right)=\frac{\sum_{s\leq t}y_{s}K_{h}\left(y_{s-1}-y\right)}{\sum_{s\leq t}K_{h}\left(y_{s}-y\right)}.$$

This will also be an estimate of the optimal predictor if the past of $y_{t+1}$ can be represented by $y_{t}$, e.g., in the possibly nonlinear autoregressive AR(1) case $y_{t+1}=g\left(y_{t}\right)+e_{t}$ for some function, *g*. In the case of the AR(p) process with $y_{t+1}=g\left(y_{t},\ldots ,y_{t-p}\right)+e_{t}$, the function *g* can be estimated, and consequently, $y_{t+1}$ can be forecasted, but one quickly runs into the curse of dimensionality as *p* increases.

To avoid the curse of dimensionality, one can use an additive model, where it is assumed that $g\left(y_{1},\ldots ,y_{p}\right)=g_{1}\left(y_{1}\right)+\cdots +g_{p}\left(y_{p}\right)$, resulting in a model:

$$y_{t+1}=g_{1}\left(y_{t}\right)+\cdots +g_{p}\left(y_{t-p}\right)+e_{t}$$

for some component functions, $g_{1},\ldots ,g_{p}$. The model can be estimated by so-called backfitting, which is thoroughly explained in [37]. There are extensions to models with mild forms of interactions between the components; see, e.g., Ref. [38]. The relevant variables can be selected by using the lasso routine, as explained in [39].

The conditional mean forecast is optimal in the least squares error sense, but it should be noted that there are alternative measures of error for forecasting, which are, in fact, often used as error measures in comparing forecasts and forecasting competitions. The most used are the mean absolute error (MAE) minimized by the median and mean absolute percentage error (MAPE). For a discussion of these and related topics, I refer the reader to [40].

Nonlinear parametric models certainly have an advantage if the data are nonlinearly generated, but, of course, they lose out to linear models if the data are linearly generated. In general, the danger of overfitting increases with nonlinear models, as can be seen with far worse examples of out-of-sample forecasts. The training (estimating of parameters) naturally takes longer due to the more involved estimation algorithms. These may be iterative, and it is not always obvious when the iteration should be stopped. Often, when they are regression-type models, they are usually not difficult to interpret.

The nonparametric forecasting algorithms are based on estimating the conditional mean and are, therefore, easy to interpret. Their scalability is a problem due to the curse of dimensionality but can partly be avoided using simplifications such as additive models.

## 4. Machine Learning Forecasts

As emphasized in the Introduction, to be able to make forecasts, it is necessary that an observed pattern has features that are preserved in time when moving into the forecasting period. Since we are dealing with time series, this seems to require some kind of stationarity in the series. However, the strictness or wide-sense stationarity of the series is not an absolute prerequisite. Multivariate time series may be nonstationary; nevertheless, it may be possible to forecast if there is a time-invariant pattern governing the nonstationarity, for example, via cointegration, where there is one or more time-invariant linear combination of the series, which results in a (possibly) multivariate stationary time series.

For a univariate time series, there could be a linear stochastic trend, which may be removed by differencing, or there could be a possibly nonlinear deterministic trend, which may be removed by a regression device. Moreover, heterogeneity may be removed using a Box-Cox-type transformation [41]. In a local Gaussian approach, further analysis is facilitated by transforming the series into a Gaussian series; see [4].

A Gaussian AR series is the series that is easiest to handle regarding prediction; everything is based on the autocorrelation function, and the optimal least squares forecast, the conditional mean, is linear and can be expressed in terms of the autocorrelation function, which, in turn, gives a complete description of the probability structure in the Gaussian case.

A key aspect of the autoregressive process is its recursive definition, where forecasts can be obtained easily by regressing using the series itself, and forecasts with a more distant time horizon can be built up recursively. The recursive property can also be built up for the ARMA and ARIMA series and, as seen in Section 2, for exponential smoothing models using Equations (2) and (3). The recursive mechanism can also be extended to seasonal forecasts.

For nonparametric and nonlinear forecasts, similar but more complicated recursive mechanisms may, in many cases, be relied on.

Common to all of these mechanisms and a prerequisite for them to work well is a training period, where the model is built up by identification, estimation, and diagnostic checking, not only in the ARIMA case but also for the exponential smoothing models. In the training period, the parameters need to be estimated, and this is similar when regarding nonlinear parametric models. The nonparametric method does not contain parameters as such, but the number of lags and the bandwidth need to be determined.

For machine learning methods to be dealt with regarding this section, the training is absolutely essential and is, in many ways, the central concept of the methodology; this is not only used for forecasting; it is used in general when finding a pattern that describes the data and where this pattern can be recognized and used in the analysis when new data are coming in. There are several ways of doing this, but I will concentrate on two methods: neural network analysis and random forest, which seem to have been the most successful machine learning (ML) forecasting methods.

### 4.1. Forecasting Using Artificial Neural Networks

Neural networks are used for a number of problems in prediction, classification, and clustering. The success and impact of neural network-like approaches in machine learning have recently been recognized by the 2024 Nobel Physics Prize awarded to John Hopfield and Geoffery Hinton. Currently, there is intense activity in this field, not the least in so-called deep neural network modeling. These are networks with several hidden layers. A detailed survey of developments up to 2015 is given in [42].

I start with a single-layer network. Assume a given data vector, x, which may be thought of as a section of a time series, $\left\{y_{1},\ldots ,y_{t}\right\}$. In a neural network approach, one is interested in transforming x via linear combinations of its components and a nonlinear activation function, often of sigmoidal form, of the linear combinations. These linear transformations represent what is called a hidden layer.

The first step in constructing the hidden layer is to form linear combinations:

$$h_{i}=w_{0}+\sum_{j=1}^{n}w_{ij}x_{j}$$

and then possibly subject these terms to an activation function. In the case of just one hidden layer, an output layer in terms of the hidden layer is given by

$$z_{i}=\sum_{j=1}^{m}w_{ij}^{\prime }h_{j}$$

followed by another possibly nonlinear function, depending on the problem. It has been shown, e.g., Ref. [43], that this system of linear combinations and activation functions is able to approximate general classes of functions. In a s-step time series forecasting problem, the output may successively be built up to obtain the forecast ${\hat{y}}_{t+s}$ at step *s* from the input set $\left\{y_{1},\ldots ,y_{t}\right\}$. When using a training set, the weights $w_{ij}$ and $w_{ij}^{\prime }$ are determined by minimizing a loss function involving $\left(y_{t+s},{\hat{y}}_{t+s}\right)$, where ${\hat{y}}_{t+s}$ is based on the output layer. The error function is evaluated for each of the samples coming in the input window. Subsequently, the output of the gradient of the error function with respect to $y_{t+s}$ is evaluated, and the weights are recomputed and updated in the direction of the gradient using stochastic gradient descent. The weights $w_{ij}^{\prime }$ for the output layer are computed first. Then, $w_{ij}$ is adjusted via the chain differentiation rule using so-called backpropagation.

The simple, one-hidden-layer forward scheme has met with considerable success. Applications such as the embedding of graphs and text processing are described in [44], with emphasis on the work carried out in [45,46]. However, it has turned out that in many problems, including forecasting, improved performance can be obtained using deep, multiple-layer networks, where the learning process is called deep learning.

A prime advantage of deep learning is that hidden units can be connected to each other recursively in time. This is taken up among several other sources in the literature in [47]. They consider a somewhat extended multivariate, one-step-ahead forecast framework, where for component *i*,

(12) $${\hat{y}}_{i,t+1}=f\left(y_{i,t:p},x_{i,t:q},s_{i}\right)$$

where $y_{i,t:p}=\left\{y_{i,t},\ldots ,y_{i,t-p}\right\}$, and $x_{i,t:q}=\left\{x_{i,t},\ldots ,x_{i,t-q}\right\}$ are exogenous variables of component *i* over the look-back window of length *p*; $s_{i}$ represents static meta-data, e.g., the location of a meteorological station, for climate data. The function *f* is the prediction function learned by the model. It contains weight functions and hidden units of the hidden layers, which, as will be seen below, can be chosen in various ways.

**Convolution neural networks (CNNs) and (TCNs):** Convolution neural networks (CNNs) [48] were originally designed for spatial data; however, they have been adapted to a time series situation. For an intermediate layer, *l*, each convolution is a causal filter. In terms of hidden vector units, it takes the form

(13) $$h_{t}^{\ell }=A\left(\left(w⋆h\right)\left(\ell ,t\right)\right)$$

with the convolution between weights and hidden units given by

$$\left(w⋆h\right)\left(\ell ,t\right)=\sum_{\tau =0}^{p}w\left(\ell ,\tau \right)h_{t-\tau }^{\ell }.$$

In (13), $h_{t}^{\ell }$ is an intermediate state at layer *ℓ*, and w is a fixed matrix of convolution weights at layer *ℓ*. The function A is an activation function, e.g., a sigmoidal function.

In a time series context, convolution neural networks are often named temporal convolutional networks (TCNs). The contrast with the spatial CNNs mainly consists of their focus on the data types and the structure of their convolutional layers. In particular, TCNs employ temporal and dilated convolutions to capture more long-range dependencies and complex temporal patterns, whereas CNNs use spatial convolutions to detect local patterns and features in images.

A basic TCN publication is [49], which is concerned with action segmentation in computer vision. The TCN algorithm has been compared to other recurrent networks like RNN and LSTM, which will be treated next. An advantage of TCNs is that, due to the parallel nature of the convolutions, they can train faster and more efficiently than LSTMs. Examples of comparisons of forecasting potential for TCN, RNN, and LSTM are [50,51]. The outcome of these investigations and similar experiments is that TCNs can compete on par with RNN and LSTM, but this somewhat depends on the data type.

A single, causal CNN layer is equivalent to an autoregressive model.

**Recurrent neural networks (RNNs):** Recurrent neural networks (RNNs) do not require an explicit fixed look-back window, as is the case for CNNs. Given the natural interpretation of time series forecasts as sequences of inputs and targets, many applications for temporal time series prediction have been developed [52,53,54]. At their core, RNN cell units contain an internal memory state, which acts as a compact memory of past information. The memory state is recursively updated from new observations at each time step.

Recurrent neural networks were originally based on workin the 1980s. Since then, a number of different RNN architectures have been developed. Just to give a flavor of these networks, I present the model equations for the three-layer (one hidden layer) Elman network. They are

(14) $$h_{t}=\sigma_{h}\left(W_{h}x_{t}+U_{h}h_{t-1}+b_{h}\right),$$

(15) $$z_{t}=\sigma_{z}\left(W_{z}h_{t}+b_{z}\right)$$

where $x_{t}$ is an input vector, $h_{t}$ is a hidden layer vector, $z_{t}$ is an output vector, W and U are parameter matrices, b is a parameter vector, and $\sigma_{h}$ and $\sigma_{z}$ are activation functions. Equations (14) and (15) show the recursive structure of the network.

The Elman network and the similar Jordan network are known to be simple recurrent networks (SRNs). There exist considerably more complicated recurrent networks with a number of layers than in CNN networks, but the idea of recursion in time is kept.

**Long short-term memory (LSTM) recurrent networks:** The long-term gradients that are backpropagated in classic RNNs can tend to zero or explode. The problem is computational. The so-called long short-term memory (LSTM) recurrent network is designed to counter this problem. LSTM units practically solve the vanishing gradient problem because LSTM units allow gradients to flow unchanged. LSTM can still sometimes suffer from the exploding gradient problem.

The vanishing gradient problem was first analyzed in a diploma thesis by Sepp Hochreiter at the Technological University of München under the guidance of Sepp Schmidhuber. After having been published as a technical report and as a conference proceeding, the full LSTM paper appeared in 1997 in Neural Computation [55]. Since then, there have been substantial and applied advances regarding the method. Now, the original LSTM paper stands with a hefty count of more than 67,000 citations. The paper has become the most cited neural network article of the 20th century, and it has been applied with considerable success to topics such as unsegmented, connected handwriting, speech recognition, machine translation, robot control, video games, and healthcare. LSTM is particularly well-suited to classifying, processing, and making predictions of time series data since there can be lags of unknown duration between important events in time series.

A common LSTM unit is composed of a cell unit, an input gate, an output gate, and a forget gate. The cell remembers values over arbitrary time intervals, and the three gates regulate the flow of information into and out of the cell.

**Transformers:** In 2017, a paper with the endearing title “Attention is all you need” [56] was published. “Attention” is here a technical machine learning term referring to a method that determines the relative importance of each component in a sequence relative to the other components in that sequence. The term has its origin in natural language processing, with the importance being represented by assigning weights to each word in a sentence (more generally, attention encodes vectors called token embeddings across a fixed-width sequence that can range from tens to millions of tokens in size). The title of the [56] paper refers to the fact that the authors were able to do away with the recursive structure of machine learning methods like RNN and LSTM and build on language processing concepts instead. This meant that parallelization could be implemented with enormous savings in processing time and could also be said to lay the foundation for artificial intelligence routines like ChatGPT.

The methodology presented in [56] has recently had a very big impact. This model of a neural network that learns context and, thus, meaning in sequential data, like the words in a sentence, has been named the transformer model (with the added fact that transformers can use parallel processing to increase speed). Transformers are, in many cases, replacing convolutional and recurrent neural networks (CNNs and RNNs) as the most popular deep learning models. Indeed, 70 percent of arXiv papers on AI posted in the last two years mention transformers. One example of further development of an aspect of transformer methodology is [57].

The technology is, in principle, valid for any sequence of data, including time series and molecular sequences and their application in medicine, leading to a large growth in use in a number of fields. The structure of transformers also allows them to be able to model long-range dependence [58]; see [59].

I will return to transformer forecasting in the section on weather forecasting—Section 6.

### 4.2. Random Forest and Gradient Boosting

Random forest and gradient boosting seem to be the main ML competitors to neural network-based models in forecasting. Both of these methods can be used both for classification and prediction. They are described well in chapters 10, 15, and 16 in [60]. Both of these methods are random tree methods and use bootstrap realizations efficiently to form new trees. Random forest uses averages of forecasts generated by independent bootstrap-generated trees and can be run in parallel. Gradient boosting runs sequentially, where the new tree model is sought to correct the errors of the previous one.

The procedure of random forest is a special case of a bagging procedure, where a final predictor or classifier is obtained by using the average of predictors/classifiers obtained by bootstrapping the original data and creating a regression tree and a forecast for each bootstrap realization. A block bootstrap has to be used in time series forecasting. The advantage of this “ensemble averaging” lies in the possible variance reduction in taking averages. The variance reduction is largest if the bootstrapped trees are independent. In the random forest algorithm, one tries to reduce dependence by using a random mechanism in the growth of each bootstrap tree.

For forecasting purposes, the tree structure used is regression trees. The range of the explanatory variables is randomly split into regions (sequentially) in time; when the tree is fully grown, the predicted value for each sub-region (possibly obtained using several splits) is given by a constant determined by a least squares algorithm on the training set for each bootstrap realization of the data. The final predicted value for a given value, x, of the explanatory variables is then obtained by taking the average of the regionalized values for the regions x belongs to for the various bootstrapped trees. Since the bootstrap realizations can be run and worked on in parallel, efficient computation time may not be too long, even for a large number of bootstraps.

Where the point of random forest is variance reduction, the purpose of gradient boosting is more directed towards bias reduction. Again, the aim is to obtain suitable regression trees. However, in this case, the bootstrap realizations are not just run in parallel. Now, a regression tree is sought by correcting the prediction error in the training period of the previous regression tree. This is a sequential procedure, and it is continued until the entire validation part of the training data set is accurately predicted or a predefined maximum number of models is reached. During this process, the weight of the original data points is changed so that points that are incorrectly predicted are given more weight. In this way, an adaptive boosting procedure is constructed. The authors of Ref. [61] developed the first version of AdaBoost, and they received the prestigious 2003 Gödel prize for their work. Compared to random forest, the results of gradient boosting are more sensitive to parameter settings during training. However, using the correct parameter settings, the user expects to obtain better test results than random forest.

The sequential procedure involved in gradient boosting may make the procedure impractical if a large set of data with many bootstrap generations is involved. Approximate methods and more time-efficient algorithms, e.g., the so-called light-GBM, have been suggested; see, in particular, [62].

### 4.3. XAI

There are a number of reports on the general scalability of machine learning algorithms; see, e.g., Ref. [63]. The same is the case for the order of training times required; a table is given in [64].

There are other ML methods that can be applied to forecasting: support vector machines, ridge regression, and the lasso process. The latter includes regularization terms to avoid overfitting. It is claimed that an ensemble method like random forest itself avoids overfitting by using the ensemble operation. Much more details can be found in [60]. A brief overview of the overfitting problem is contained in [65].

As is mentioned repeatedly in this paper, the largest problem of machine learning methods is their black box nature. Both neural network and random forest prediction algorithms produce forecasts, but they are not simply expressible in terms of the input variables (features) of the model. It is understandable that skepticism arises towards machine learning when it is difficult to understand or (intuitively) explain how each feature contributes to the model. The situation is very different from forecasts formed from generalized linear regression models, where one can find a natural measure of how each input variable contributes to the forecast. Is it possible to find similar measures for the contribution of each input feature in a machine learning context?

Work on this problem has actually started and has, in fact, spawned a whole new discipline: “Explainable artificial intelligence”, XAI, where the aim is to “explain” black box models. A recent survey of this field is given in [66], where the problem is discussed broadly and partly from a philosophical point of view. There are also papers devoted to this problem in special research areas, e.g., opening the black box in applications in genetics, such as in [67], where three general techniques are being used to probe the kinds of changes in the output are being caused by the alternative weighting of structures in the model. Sensitivity analysis can be used to examine the effects of modifying the input features. The model in [68], which is treated in Section 5.3, was tested by undergoing such sensitivity analyses. A final strategy mentioned in [67] is the surrogate strategy, where the black box model is sought to be replaced by a simpler, more interpretable model, which still provides a good approximation.

There are also techniques that seek to attribute a concrete value to the importance of each input feature, thus constituting, in a sense, a generalization of the importance measure of each input variable in a generalized linear regression model. The most used technique is probably the SHAP value, which is related to the Shapley value in competitive games. The Shapley value was introduced by Lloyd Shapley in 1951, see [69], for which he was later awarded the Nobel Prize in economics. To put this into our present context (as in [70]), let *F* be the set of all features. The Shapley method, in principle, requires retraining a model on all feature subsets, S⊆F. The method assigns an importance value to each feature, *i*, that represents the effect of the model prediction of including that feature. To compute this effect for the *i*th feature, a model, $f_{S\cup i}$, is trained with that feature present, and another model, $f_{S}$, is trained with the feature withheld. These predictions from the two models are compared to the current input forming the difference $f_{S\cup i}\left(x_{S\cup i}\right)-f_{S}\left(x_{S}\right)$, where $x_{S}$ represents the values of the input features in the set *S*. Since the effect of withholding a feature depends on the other features in the model, the preceding differences are computed for all possible subsets, S⊆F−i. The Shapley values are then computed and used as feature attributions (importance). They are weighted averages of all possible differences

$$\phi_{i}=\sum_{S\subseteq F-i}\frac{|S|!(F-|S|-1)!}{F!}\left[f_{S\cup i}\left(x_{S\cup i}\right)-f\left(x_{s}\right)\right]$$

where |S| is the number of elements in *S*.

In [70] (also see [71]), the Shapley values are integrated in an additive regression-type framework to create SHAP values (SHapley Additive exPlanations). This framework also serves as a common framework for five other explanation models, with LIME (local interpretable model-agnostic explanations) described in [72] among them. Some stability issues are highlighted in [73]. Based on the unification attained in [70], the authors indicate that the new methods show improved computational performance and/or better consistency with human intuition than previous approaches. As an example among numerous applications of SHAP in explaining machine learning models, real hospital data are examined in [74].

It should be remarked that the above examples mark only the beginning of the problem of opening the black box for machine learning models. A rather critical evaluation of XAI is contained in [75]. Moreover, rather limited attention has been given to this issue in the forecasting competitions treated in the next subsection. One reason for this is probably due to the rather late appearance of XAI methods. There is surely a need for competitions that compare XAI possibilities for various models.

### 4.4. Statistical vs. ML Methods: Evidence from Forecasting Competitions

A number of forecasting competitions have been arranged to find the “best” forecasts and to evaluate statistical forecasts against ML forecasts and simple models vs. more complicated ones. Spyros Makridakis, in particular, has organized a number of forecasting competitions, starting in 1982 with the M1 competition and then continuing with the M2–M6 competitions, with the results published in 1982, 1993, 2000, 2020, 2022, and 2024 (see below for exact references). The scope has been somewhat varied, and so has the number of participants and the number and versions of methods represented. For the most recent ones, major ML and statistical methods have been included. In the M1 competition, the number of time series sought to be predicted was 1001; in M3, it was 3003, and in M4, it was 100,000. A history of forecasting competitions up to 2020 is given in [76]. The forecasting competitions described by the forecast organizers themselves can be found in [6,77,78,79,80,81].

The general tendency in the course of these competitions is that ML methods have been more successful. In the first competition, simple statistical models of the exponential smoothing type did better than the more complex ARIMA models. In M3 in the year 2000, several ML methods did partake, but the winner was the theta method described in Section 2, a relatively simple parametric extension of exponential smoothing -type models. The M1–M3 competitions had a relatively small number of time series available for prediction. The M4 competition represented a very significant extension, featuring 100,000 time series, and it is also (to date) the competition with the most general types of series; it was also the first where prediction intervals were evaluated in addition to point forecasts. It seems reasonable to give a brief review of that contest. The time series differed in sampling frequency: yearly, quarterly, monthly, daily, and hourly, with about half of the series sampled on a monthly basis. The series was taken from the following areas: business, economics, industry, demographics, education, labor and wages, government, households, bonds, stocks, insurance, loans, real estate, transportation, natural resources, and the environment. The sheer amount of time series and their spread is, of course, commendable, but, at the same time, this could be discouraging for the competitors due to the amount of labor required. There was also a relatively large number of dropouts because the forecasting teams were discouraged when they compared their results to simple benchmark forecasting methods supplied by the organizers. Nevertheless, 61 forecasting methods ended up being represented.

The length of the time series varied considerably, with the minimum number of observations in the training set being 13 for yearly, 16 for quarterly, 42 for monthly, 80 for weekly, 93 for daily, and 700 for hourly series. However, on average, the length of the series was much longer than the series in the M3 contest. The forecasts were evaluated in several ways, mostly based on relative absolute deviation, with an average taken over forecasting horizons, which varied from 3 for yearly data to 48 for hourly data.

The winner of the competition was Slake Small, a data scientist at Umber Technologies. He mixed exponential smoothing-type models with RN recursive deep learning methods, which are described in more detail in [82]. His hybrid method perhaps represented a main result of the competition, namely the improved numerical accuracy of combining forecasting methods and the superiority of a hybrid approach that utilizes both statistical and ML features.

Combining has long been considered a useful practice in the forecasting literature, going all the way back to the classical paper of [83] for purely statistical methods and up to the ensemble methods for random forest and gradient boosting. Intuitively, it is understandable that the combining principle can lead to improvements, as different methods pick up different forecasting potentials of the data, but the exact theory that explains the improvement is more scarce; see, e.g., Ref. [84]. More specifically, for the M4 competition, there was only 1 pure point forecast statistical method among the top 10 most accurate methods, while all of the 10 least accurate methods were either purely statistical or purely ML. Similar results were obtained for interval forecasting.

It seems that most of the methods were applied to individual series, with only a few attempts to exploit multiple series to extrapolate the individual ones. Similarly, exogenous variables do not seem to have been used (possibly not the point of the competition and perhaps not so easily available).

Another issue not dealt with by Makridakis et al. and by other similar contests is that there was no emphasis on so-called black swans, i.e., in the evaluation of the forecasts, it was not registered whether a forecasting method in some cases did disastrously badly; this would be missed in an evaluation based on average performance over the series. The “black swans” may, in some cases, be caused by thick-tailed distributions [85]. The organizers remark that this phenomenon was outside the scope of the M4 competition but stated that another forecast competition is needed for closer examination to analyze the implications of black swans for risk management and decision-making.

A total of five ML methods, four pure and one combination of solely ML methods, did not do well. These results were in agreement with [24]. However, it could be possible that this is partly due to the ML methods used in the competition. For example, it does not seem that the best-developed random forest or gradient boosting processes participated.

These types of criticism (partly pointed out by the organizers themselves) and some additional ones are taken up in [86]. They refer to the results of a recent competition of 145,000 series on web traffic data run by the Kaggle corporation and hosted at about the same time. The time series in the Kaggle data had a much higher spectral entropy (defined by $-\int_{-\pi }^{\pi }f\left(\lambda \right)\log f\left(\lambda \right)d\lambda$, where f(λ) is the spectral density of the series). This means that the series were noisier and more difficult to predict. Further, many more series had (much) shorter time intervals between the samples. For the Makridakis data mentioned above, the emphasis is on monthly data (48%), whereas only 0.4% were weekly, 4.2% daily, and 0.4% hourly.

In [86], Fry and Brundag state that at Google, as is the case with many other net-based companies, there is an increasing degree of situations where one has to deal with short time intervals, e.g., even 5 min intervals, and sometimes irregular time intervals. For these data with higher spectral entropy, it has turned out that ML methods have been doing increasingly well, and in [86], Fry and Brundage state that ML methods have, in fact, been a game-changer for this kind of forecast. At the same time, they reiterate that more emphasis should be put on multivariate and hierarchies of time series, as well as on predicting extreme events. They state that perhaps more attention should be put on predicting higher quantiles in a forecast distribution than on producing a single-point forecast.

Perhaps as a reaction to this, a new M5 forecast competition was launched [80], which focused on retail sales forecasting applications and used real-life hierarchically structured sales data with intermittent and erratic characteristics. The competition attracted many participants who were eager to experiment with effective forecasting solutions in real-life situations faced by numerous retail companies on a daily basis. Many of the series in the competition were characterized by intermittency, involving sporadic unit sales with many zeroes.

The data set used comprised the unit sales of 3049 products sold by Walmart in the USA. The data were organized in the form of grouped time series aggregated based on their type (category and department) and selling locations (stores and states), with a total of 42,480 series in 12 cross-sectional aggregation levels. As a performance measure, a scaled least squares criterion averaged over several forecasting horizons was used.

Again, simple methods like exponential smoothing did fairly well. However, this time, they were clearly beaten by the best ML methods. The light-GBM version of gradient boosting mentioned in Section 4.2 turned out to have superior forecasting capabilities compared to all other alternatives, and it was used in practice by all of the 50 competitors. This is consistent with the claim of [86] regarding the superiority of ML methods for this and similar types of series. Somewhat curiously, the first prize was won by a senior undergraduate at Kung Hee University of South Korea with the team appellation YJ.STU. He used an equal-weighted average of various light-GBM algorithms.

The most recent of the Makridakis forecasting competitions is M6 [81]. Participants were asked to forecast 100 financial assets and to make corresponding financial investments. Surprisingly, there was virtually no correlation between the best forecasts and the best investment decisions; it turned out to be very difficult to beat the standard S&P market index. The authors do warn that the participants to a limited degree carried out the study according to the intentions of the organizers. It was intended that the competitions should have 12 non-overlapping submission periods, with each 28 days after the previous one. As a result, the competition lasted for almost one year. However, only a small percentage of the teams updated their forecast regularly during the duration of the competition. Many teams simply submitted once or twice at the very beginning of the competition. Again, a winning team was directed by a graduate student.

It is satisfying that ML techniques have the potential to obtain better forecasts, and they do increase the tool box of forecasters. However, there are reasons for some reservations and criticisms directed towards ML methods. A primary reason, as mentioned, is that they are black box forecasts based on pattern recognition of data types. Some attempts, see, e.g., Ref. [68] (who obtain their best results using random forest routines), have been made to look into this by comparing particular cases of benchmark forecasts, changing the parameters of an ML forecast, and seeking to explain the corresponding changes in the forecasts. See Section 5.3 for further mentions of this paper. Basically, ML methods are not immediately interpretable, as e.g., an AR time series model; however, as indicated in Section 4.3, attempts to open the black box have started to emerge. Moreover, another distinct difference is the lack of theoretical results on the asymptotics of ML methods. The beginning of theoretical development may be represented in [87].

Summing up, regarding the results from the Makridakis competitions and other similar competitions like the Kaggle competitions, there seems to be a definite trend where the ML forecasts are improving, and at least, in some areas as those with high spectral entropy are superior to statistical forecasts. However, limitations of these competitions, other than the black box criticism of ML methods, have been pointed out. For example, they have essentially been carried out for univariate series. They started out by using more or less pure point forecasts. Interval prediction has been increasingly included, but there is still a lack of clear results on forecasting distributions and extreme quantiles. Moreover, there is a lack of systematically evaluating multivariate forecasts, the influence of exogenous variables, and the effect of nonstationarity on multivariate systems. The next section will be devoted to some of these themes.

## 5. ML vs. Statistical Models in Some Other Situations

Based on the contents and criticisms of the Makridakis competitions, it can be stated that they do not constitute a complete coverage of all aspects of the forecasting problem. In this section, I will look briefly and somewhat unsystematically at some of the missing aspects. Each of these subject areas would benefit from a systematic study in a framework of M-type forecast competitions. Ordinary statistical models seem to still dominate in these areas, but there has been some recent work on ML methods.

### 5.1. Probability Forecast

Sometimes, one is not satisfied with an interval for measuring forecast error and would like more detailed information in terms of forecast distribution. This is easily obtained for a linear Gaussian model, e.g., an AR(p) model, as defined in (4), where the forecast distribution is obtained as $N(a_{1}y_{t-1}+\cdots +a_{p}y_{t-p},\sigma_{e}^{2})$, where $\sigma_{e}^{2}$ is the variance in the error process $\left\{e_{t}\right\}$. Similar reasoning can be put forward for an additive model with a Gaussian error process. In a more general situation for a multivariate series $\left\{y_{t}\right\}$, in principle, kernel estimates can be used to obtain estimates of the conditional distribution

(16) $$p\left(y_{t+1}|\left(y_{t},\ldots ,y_{t-p}\right)\right)=\frac{p(y_{t+1},y_{t},\ldots ,y_{t-p})}{p(y_{t},\ldots +y_{t-p})}$$

but the curse of dimensionality comes into play for moderate dimensions of {y} and for moderate orders, *p*. In addition, the denominator may be close to zero in the estimates of the tail, which may cause instability.

A possible alternative may be to use local Gaussian estimates, as detailed in [4], in particular, chapters 9 and 10 on local Gaussian density and conditional density estimation; see also [88].

A very recent conditional probability model coupled with the theory of diffusion processes has recently obtained very good results in weather forecasting; see the end of Section 6.

In dealing with forecast distributions, one meets the fundamental problem of how to compare them. When is one distribution significantly better than another? This problem is equally valid for point forecasts and has been discussed in the classical paper [89]. They introduce a process for testing whether one predictor is significantly better than another. The authors of Ref. [90] treat a similar problem for probability forecasts.

Actually, Gneiting and colleagues have discussed a number of fundamental questions related to probability forecasting; see, e.g., the editorial [91] by Gneiting and [92]. In particular, he and his coauthors examine the process of forecasting extreme events [93] and combining probability forecasts in [94].

The ML literature on probabilistic forecasting is more sparse. One relatively recent contribution is [95]. The authors concentrate on finding forecasts of quantiles, which is a very relevant expression of the probability distribution, perhaps in economics and finance in particular. Several versions of a DeepAR quantile estimate have done well compared to more traditional benchmark estimates when compared to those forwarded already in [96].

A spline approach coupled with an RNN, see Section 4.1, is advocated in [97], whereas the authors of [98] use a copula approach.

### 5.2. Volatility Forecasting and a Machine Learning Example

Volatility is an all-important concept in the analysis of financial risk. Rob Engle received the Nobel Prize for his contributions to this field, in particular, in [99,100]. The resulting models are the ARCH/GARCH models, which are now well represented in any textbook in econometrics. The GARCH model for the instantaneous conditional variance $\sigma_{t}^{2}$ is given by

$$\sigma_{t}^{2}=\omega +\sum_{i=1}^{p}\alpha_{i}\varepsilon_{t-i}+\sum_{j=1}^{q}\beta_{j}\sigma_{t-j}^{2}$$

where ω is a constant term, and $\left\{\varepsilon_{t}\right\}$ is the heteroscedastic error term. There are many specialized versions of the GARCH model, e.g., EGARCH, that take care of the leverage effect of volatility,

$$\log\sigma_{t}^{2}=\omega +\sum_{i=1}^{p}(\alpha_{i}\varepsilon_{t-i}+\gamma_{i}{\left(|\varepsilon \right|}_{t-i}-E|\varepsilon_{t-i}\left|)\right)+\sum_{j=1}^{q}\beta_{j}\log\sigma_{t-j}^{2}$$

where the new parameter $\gamma_{i}$ captures the asymmetric impact of news with negative shocks. An alternative class of volatility models is the so-called SV (Stochastic volatility) models; see, e.g., Ref. [101].

The increased use of high-frequency data has led to the introduction of a new instantaneous variance concept: realized volatility. High-frequency data can really be looked at as a process where events take place continuously in time. A simple model for the price process $P_{it}$ for a financial asset *i* is given by

$$d\log P_{it}=\mu_{i}+\sigma_{it}dW_{t}$$

where $\mu_{i}$ is the drift, $\sigma_{it}$ is the instantaneous volatility, and $W_{t}$ is the standard Brownian motion. In practice, a discrete approximation is used. The mid-price return for asset *i* during the time interval (t−1,t] is considered given by

$$r_{it}=\log\left(\frac{P_{it}}{P_{i,t-1}}\right).$$

Refs. [102,103] argued that the sum of squared intraday returns is a consistent estimator of the unobservable theoretical integrated variance, $\int_{t-h}^{t}\sigma_{is}^{2}ds$, where *h* is the look-back horizon, such as 10 min, 30 min, 1 day, etc. The realized volatility can be based on the logarithm (to reduce the impact of extreme values). Ref. [104] specifically defined the following during a period (t−h,t]:

(17) $$RV_{it}^{\left(h\right)}=\log\left(\sum_{s=t-h+1}^{t}r_{is}^{2}\right).$$

with a 5 min return interval.

Previously, forecasting volatility was based on parametric models. Very recently, the authors of [105] used ML methods and set up a simple forecasting comparison between several models. Among other things, they based themselves on (17) but with a 1 min return interval. Ref. [105] argues that, generally speaking, there are two broad families of models used to forecast daily volatility: (i) GARCH and SV models that employ daily returns and (ii) models that use daily RVs. Previous well-established studies have shown that due to the utilization of available intraday information, daily RV is a superior proxy for unobserved daily volatility when compared to the parametric volatility measures generated from the GARCH and SV models of daily returns [103,106,107].

Two questions can be asked. It has been observed that in asset markets, there seems to be a common intraday structure that is shared among the volatility of the assets. It is termed commonality. The first question is, can intraday commonality structure be utilized in improving volatility forecasts? The second question has to do with the main theme of the present paper: Can forecasts based on parametric models be improved upon by ML methods?

Both questions are considered in [105] in their handling of data. They use a data set composed of the top 100 components for the S&P index for the period between 1 July 2011 and 30 June 2021. After filtering away the shares for which data did not exist for the entire period, 93 stocks were kept. The average realized volatility commonality for the market is

$$RV_{M,t}^{\left(h\right)}=\frac{1}{n}\sum_{i=1}^{n}RV_{it}^{\left(h\right)}$$

and because a measure of the commonality is used, the $R^{2}$ value of the regression is

$$RV_{it}^{\left(h\right)}=\alpha_{i}+\beta_{i}RV_{M,t}^{\left(h\right)}+\varepsilon_{it}$$

which is inspired by prior studies, as in [108,109].

The data were subjected to a number of methods, both parametric and ML, and the outcomes were compared for a one-data set volatility forecasting competition.

The two questions were treated for a number of models for instantaneous volatility and its development in time. Among the models included were seasonal ARIMA, HAR models (cf. [110]), ordinary least squares, least absolute shrinkage, XGBoost (essentially a gradient boost technique, as described before in Section 4.2), multilayer perceptron (MLP) (a multilayer feedforward neural network), and long short-term memory (this is the LSTM modeling routine described earlier).

For these models, and with the data set divided into a training period and a validation period, an extensive data analysis and comparison was carried out. Consistent with the M5 competition, the ML methods, in particular, the neural network-based methods MLP and LSTM, gave the best volatility forecasts. Moreover, for most models, taking advantage of the commonality information leads to better forecasts. Finally, contrary to the M6 competition, the forecasting evidence seems to be consistent with the results obtained in constructing investment portfolios based on forecasts. Much more detail and a number of related references can be found in [105]. It should also be mentioned that in the study of forecasting the volatility of cryptocurrencies [111], it was found that forecasts can be improved by combining GARCH-type models with ML forecasts.

As remarked upon in Section 1 and Section 4.4, an alternative measure of uncertainty of data is given by entropy. In the Kaggle competition referred to in Section 4.4, the importance of the entropy measure was stressed. The use of entropy in financial time series (also pointing out the relationship to volatility) has been mentioned in several recent publications.

In [112], structural entropy is introduced, and the new measure and volatility are remarkably correlated for an asset price series; see also [113,114]. Related to [111] is [115], and both deal with cryptocurrency. Finally, Ref. [116] used entropy in the description of sudden shocks, whereas [2] studied entropy-corrected geometric Brownian motion.

### 5.3. Multivariate Forecasts, Including Exogenous Variables, Panels, and Cointegration

While multivariate ML methods have crept up relatively recently, multivariate data and models have been used for a long time in multivariate statistical analysis. The vector autoregressive model, VAR, is

(18) $$y_{t}=A_{1}y_{t-1}+\cdots +A_{p}y_{t-p}+e_{t}$$

where $\left\{y_{t}\right\}$ is a vector data process, $A_{i},i=1,\ldots ,p$ are autoregressive matrices, and $\left\{e_{t}\right\}$ is a vector innovation process; this has been used extensively in econometrics, finance, and other fields. This pure autoregressive model may be complemented by a set of exogenous variables $\left\{x_{t}\right\}$, resulting in the VARX model. Such multivariate models were largely not present in the Makridakis competitions, but clearly, they can be important in forecasting. If there is dependence between the components of a vector process, the intuitive use of the dependency information should lead to improved forecasts of the individual series.

So, are there any systematic forecasting exercises where classical autoregressive-type models are compared with ML methods? It seems that, thus far, as for the volatility case, there are only specific examples of such comparisons, and these mostly pertain to a situation where a single variable time series, $\left\{y_{t}\right\}$, depends on *q* explanatory variables, $\left\{x_{1t}\right\},\ldots ,\left\{x_{qt}\right\}$. One such example that gives a good illustration is [68], where forecasting the inflation of the US consumer price index was studied. The possible drivers of inflation are taken from the so-called FRED-MB database, which is a large monthly database designed for analysis in data-rich environments. The data period is from January 1960 to December 2015, with 672 monthly observations. There are 122 explanatory variables. All variables were transformed to achieve stationarity.

The benchmark models include univariate autoregressive, random walk, and unobserved component stochastic volatility models. A number of other models, including ML models, were tried. Forecasts were computed for 3-, 6-, and 12-month horizons. The authors found that, overall, random forest outperformed the other methods (it does not seem that the LSTM algorithm was included in the study, though). As mentioned in Section 4.4, the authors attempted to obtain more insight into the black box constituting random forest in this case.

An example where LSTM does well compared to other methods in multivariate time series forecasting is in [117]. The LSTM method is extended with a so-called attention mechanism (see Section 4.1) for the general use of the attention mechanism under the description of transformers. However, random forest-type forecasts were not included in the application of forecasting to solar energy data, among other things.

An example of the use of explanatory variables regarding the climate is presented in [118]. Temperatures in five Chinese cities were sought to be explained using dew point temperature, relative humidity, air pressure, and cumulative wind speed data. In a comparison with the lasso method and ordinary least squares, LSTM methods performed best. Random forest and gradient boosting methods were not included.

Finally, Ref. [119] is a paper on the prediction of soil movement, where multivariate methods did better than univariate versions, and LSTM-type forecasts were outperformed by general parametric statistical models like seasonal AR models. There seem to be relatively few data samples in this investigation.

In case there are many time series, panel models are traditionally used instead of VAR models. A quite general linear panel model can be stated as

(19) $$y_{it}=\alpha_{i}+\mu_{t}+\beta_{i}^{\prime }x_{it}+u_{it}$$

where $y_{it}$ is the dependent variable for member *i* of the panel at time t, with 1≤i≤N and 1≤t≤T, and where the apostrophe (’) stands for transposed. Moreover, $x_{it}$ is a vector of explanatory variables, for which the influence on $y_{it}$ is determined by the coefficient vector $\beta_{i}$. The scalars $\alpha_{i}$ and $\mu_{t}$ are specific panel member and time effects, respectively. Finally, $u_{it}$ is a residual term. The parameters $\alpha_{i}$, $\mu_{t}$, and $\beta_{i}$ may be deterministic (fixed effect) or random.

Model (19) is sometimes termed a static panel, whereas the term dynamic panel is reserved for the model

(20) $$y_{it}=\alpha_{i}+\mu_{t}+a_{i}y_{i,t-1}+\beta_{i}^{\prime }x_{it}+u_{it}$$

with an explicit dependence on past *y*-s. Of course, both models may be generalized to contain higher-order time lags for the *y* and x variables. A panel may also be unbalanced, where the number of observations, *T*, may be allowed to depend on *i*, $T=T_{i}$, but I will not be concerned with these generalizations. Actually, I will introduce several restrictions of (19) and (20). First, one or more of the terms in these equations may be omitted. Second, if the parameters are not allowed to depend on *i*, the panel is said to be homogeneous, whereas dependence on *i* leads to a heterogeneous panel. In the latter case, it is simplest to treat the case where the parameters are random and have the same distribution for each *i*. Often, independence over *i* is added as an extra assumption, and the task of estimation may be reduced to simply estimating the mean of the parameters. In general, averaging techniques can be used for handling heterogeneous effects, while differencing and cointegration (see below) may represent instruments for treating nonstationary effects.

The theory of panels has concentrated quite a lot on inference in parametric models. However, the potential use of panels in forecasting is obvious. A recent survey of panel forecasting using relatively traditional linear panel models is given in [120].

Nonlinear models do exist, and recently, neural networks have been included in the modeling of nonlinearities; see [121]. The authors find significant forecasting gains regarding feedforward neural networks when compared to linear panels and nonlinear time series models when examining predictive power for new COVID-19 cases. They were also able to find some of the asymptotic properties of their method using arguments originally developed in [87].

For several forecasting methods, there is no special device for taking care of nonstationarity. For traditional multivariate ARIMA models, however, individual differencing of the components was the favorite tool; this has also been used for panels. With the introduction of cointegration models, this has changed, and an initial step would consist of looking for (linear) cointegrating relationships; the cointegrating VAR model could be restated as a so-called error correction model.

Let $\left\{y_{t}\right\}$ be a vector of time series variables that are (individually) of the I(1) class; i.e., they can be made stationary using differencing. Then, by reparametrizing, one obtains the VECM (vector error correction model)

(21) $$\Delta y_{t}=\alpha \beta^{\prime }{\left[1,t,y_{t-1}^{\prime }\right]}^{\prime }+\Gamma_{1}\Delta y_{t-1}+\cdots +\Gamma_{k-1}\Delta y_{t-k+1}+\Phi d_{t}+\mu +\varepsilon_{t}$$

where the matrices α and β have a cointegration framework interpretation of Equation (18), and $\Gamma_{1},\ldots ,\Gamma_{k-1}$ and Φ are coefficient matrices; μ is a coefficient vector, $d_{t}$ is a vector containing the stationary, I(0), variables, and $\varepsilon_{t}$ is a process of residual terms often assumed in the model to be iid normally distributed. See, e.g., Refs. [122,123].

Is it possible to couple ML methods with the concept of cointegration? An example of this can be seen in [124]. Their first step is to include a nonlinear term in the error-correcting model, for example, a STAR model, as introduced in Section 3. Then, the linear part is subtracted from the full model, and the nonlinear part is sought to be re-estimated using random forest. In this way, a mixed random forest cointegration model is obtained. The model is applied to weekly forecasts of a gasoline price process cointegrated with international oil prices and exchange rates. This mixed model provided better forecasts than a purely parametric model.

It should be mentioned that deep learning forecasts have also been used in portfolio construction for cointegrated stocks. The interested reader is referred to [125].

### 5.4. GARCH-Type Schemes for Integer and Continuous Time Series

Lately, there has been an increasing interest in integer time series. These are time series consisting of counts with a low number in each time period, so a continuous approximation of the counts does not work well.

There are two main classes of integer time series models: the INAR class and the INGARCH model. In the INAR model, a convolution trick is used to obtain integer time series that follows an AR model. In the INGARCH model, a recursion is used on a parameter of an integer-based distribution. The use of ML methods for both classes is very limited (to my knowledge). A recent article for INAR fuzzy series is [126]. Negative binomial likelihood analysis with ML was briefly mentioned in [52]; see also [127].

Here, I will place emphasis on INGARCH models due to their many generalizations to multivariate models and continuous time models with connections to so-called Hawkes processes and multivariate time series in finance. The most straightforward INGARCH model is Poisson autoregression. Earlier attempts are represented, mainly using second-order theory, in [128] and, more recently, in [129]. A more thorough introduction to inference theory and ergodicity is given in [130], focusing on Poisson autoregression.

Let $\left\{y_{t}\right\}$ be an integer time series, and let $F_{t}^{y,\lambda }$ be the σ-algebra generated by $\left\{y_{s},s\leq t,\lambda_{0}\right\}$. Here, $\left\{\lambda_{t}\right\}$ is the time-varying random intensity of a Poisson process $\left\{y_{t}\right\}$. The first-order Poisson autoregressive model is given by the following GARCH-like recursion:

(22) $$y_{t}|F_{t-1}^{y,\lambda }∼\mathrm{Poisson}\left(\lambda_{t}\right)\;\lambda_{t}=d+a\lambda_{t-1}+by_{t-1}$$

where *d*, *a*, and *b* are the parameters to be estimated. Probabilistic properties and an asymptotic theory are established in [130]. It is obvious how forecasts can be developed in this scheme, and forecasts were used in tests of fitting financial data in [130].

A number of generalizations have been developed to cover higher-order models and nonlinear models, like threshold and exponential autoregressive considerations. Further, logarithmic intensity, alternative integer distributions, like the negative binomial in the recursive scheme, and multivariate cases have been examined. Some selected references can be found in [131]. Again, to my knowledge, machine learning methods have not yet been used much, although one could easily think of situations where such an approach could be useful, in particular, for high-dimensional and nonlinear integer time processes.

A generalization to continuous time regarding the GARCH-like structure can be found in [132,133] for self-excited jump processes. Let $\left\{T_{n}\right\}$ denote the times of events in a point process. We introduce the counting process associated with the point process,

$$N\left(t\right)=\sum_{T_{n}\leq t}1_{T_{n}\leq t}$$

which counts the number of events up to time *t*. The corresponding σ algebra is $F_{t}^{N}$, generated by {N(s),0≤s≤t}. A stochastic intensity process, $\left\{\lambda_{t}\right\}$, corresponding to (22) can now be generated using a GARCH-like stochastic differential equation:

(23) $$d\lambda_{t}=\alpha \left(\lambda_{0}-\lambda \left(t\right)\right)dt+\beta dN\left(t\right).$$

The model in (23) can be extended to the multivariate case, where one component may excite another component. Applications to financial data are presented in [133]. The authors also briefly discuss nonlinear models of this type. In the linear case, the models generated by recursion can be considered as a parsimonious form of Hawkes processes, where the intensity is generated by a continuous moving average mechanism with λ(t)=∫g(t−s)dN(s) for some function, *g*. These processes were originally introduced in the study of the intensity of earthquakes but were later used in finance; see [134].

The question of predicting the intensity process is an actual one. It is, again, straightforward to use the recursive equation itself, but can machine learning methods be used? Some ideas are presented in [135].

The intensity process in these applications replaces a time-varying parameter by using a stochastic process. A quite general framework for time-varying parameters is developed in [136]. The mechanism used to update the parameters over time is the scaled score of the likelihood function. The authors remark that this approach provides a unified and consistent framework for introducing time-varying parameters in a wide class of nonlinear models, with the GARCH model, autoregressive conditional intensity cases, and types of Poisson count models among them. Numerous follow-up papers have addressed the application of such models to forecasting. A recent survey paper is [137]. I am not aware of machine learning forecasts for this model.

In this section, our final recursive system will be a recursive system for a dynamic network. With the increasing use of the internet and Big Data, the analysis of large networks is becoming more and more important. There is a wide field of applications, which ranges over such diverse areas as finance, medicine, and sociology, including criminal networks. A broad overview can be found in a recent book [138]. More foundational problems are covered in [139].

Both research and applications have been overwhelmingly concentrated on static networks. However, a change seems to be taking place presently since the static assumption is an untenable one in many practical cases. As time goes on, new nodes are added to the network, others vanish, and the strength of the connection between nodes may be changed or even severed. This realization has led to a rapid increase in interest in dynamic networks, noted, for instance, in the last section of [140], where a substantial portion of the following material is taken from.

A recent example of rigorous statistical modeling regarding a dynamic network is [141]. The authors modeled the network structure by using a network vector autoregressive model. This model assumes that the response of each node at a given time point is a linear combination of (a) its previous value, (b) the average of connected neighbors, (c) a set of node-specific covariates, and (d) independent noise. More precisely, if *n* is the number of nodes, let $y_{it}$ be the response collected from the *i*th subject (node) at time *t*. Further, assume that a *q*-dimensional node-specific random vector $x_{i}={\left(x_{i1},\ldots ,x_{iq}\right)}^{\prime }\in R^{q}$ can be observed. Then, the model for $y_{it}$ is given by

(24) $$y_{it}=\beta_{0}+x_{i}^{\prime }\gamma +\beta_{1}n_{i}^{-1}\sum_{j\neq i}a_{ij}y_{j,t-1}+\beta_{2}y_{i,t-1}+\varepsilon_{it}.$$

Here, $n_{i}=\sum_{j\neq i}a_{ij}$ represents the total number of neighbors of the node, $v_{i}$, associated with $y_{i}$, so it is the degree of $v_{i}$. The term $x^{\prime }\gamma$ is the impact of covariates on node $v_{i}$, whereas $n_{i}^{-1}\sum_{j\neq i}a_{ij}y_{j,t-1}$ is the average impact from the neighbors of $v_{i}$. The term $\beta_{2}y_{i,t-1}$ denotes the standard autoregressive impact. Finally, the error terms, $\left\{\varepsilon_{it}\right\}$, are assumed to be independent of the covariates and are iid normally distributed.

There are a number of differences between the network vector autoregression modeled using Equation (24) and a full dynamic network embedding. Even if the autoregressive model does introduce some (stationary) dynamics in time, the parameters are static; i.e., no new nodes are allowed, and the relationship between them is also static, as modeled by the matrix $A=\{a_{ij}\}$. From this point of view, as the authors of [141] are fully aware, the model (24) is not realistic in terms of the dynamics that take place in practice for many networks. On the other hand, the introduction of a stochastic model that can be analyzed using traditional methods of inference is to be lauded. A worthwhile next step is to try to combine more realistic models with a stochastic structure (e.g., regime-type models for the parameters, as in [142]. The hope is that it will be amenable to statistical inference. Ref. [143] treats dynamic networks with a fixed number of nodes but only where the dependence between nodes is modeled by a doubly stochastic matrix.

For some very recent contributions to network autoregression, see [144] and the references therein. In [145], the authors consider integer-valued network variables and analyze linear and log-linear Poisson autoregressive networks. This is motivated by the fact that many net variables take discrete values. In [146], nonlinear models and tests for linearity are introduced.

An interesting recent publication is [147]; the authors study time series networks for both integer and continuous-valued data. The nonlinearity is provided by a smooth link function. The authors studied stability conditions for such multivariate systems and developed quasi-maximum likelihood estimates when the dimension of the network was increasing. They also develop linearity tests.

Again, forecasts can, in principle, be formed directly from the recursive equations. I do not know of instances where the dynamic network model and ML have been applied to the same data set.

## 6. Weather Forecasting

I will finish this paper by looking at a problem where the main thrust of the existing forecasts depends on a (numerical) physical model, namely weather forecasting. What are the interactions, if any, between this numerical physical model and ML techniques like neural networks and random forest? Is it possible to completely do away with numerical weather forecasting (NWP) and base a weather forecast entirely on ML? NWP is very time-consuming and requires extensive computer resources. For the fastest and most effective ML methods, one might think that there could be a gain in processing time, and, of course, the hope is that, ultimately, ML may increase the accuracy of weather forecasting.

Manual NWP was first attempted in [148] in Britain in 1922, and early computer-aided weather forecasts were produced in the 1950s by a group led by John von Neuman at the Princeton Institute of Advanced Studies; see [149]. The early history is also outlined in [150,151]. Modern weather prediction relies extensively on massive numerical simulation systems that are routinely operated by national weather agencies all over the world. Statistical analysis plays a major part in the stepwise procedure that NWP consists of.

In order to perform NWP, a variety of observations regarding atmosphere, land, and ocean are collected all over the world, creating a global observation network, not the least in terms of satellite measurements. Although millions of different direct and indirect measurements are obtained each day, these raw data must be transformed and refined before they can be input into the numerical weather model. This stage is called data assimilation (DA). The data are then projected into a discrete model grid (interpolated in time) and adjusted to be consistent in terms of state variables (e.g., temperature, pressure, wind, etc.). DA also takes care of measurement errors, such as biases between different spatial instruments. The obtained initial Earth system after the DA step constitutes an optimized estimate of the real conditions, cf. [152].

Given these initial conditions, the core of the NWP model consists of numerically solving the coupled partial differential system (the Navier-Stokes equations) describing the atmosphere in terms of momentum, mass, and enthalpy.

The Navier-Stokes equations for fluid motion, v, underlying atmospheric flow are the following, cf. [153], p. 14:

(25) ∇·v=0

(26) $$\rho \left(\frac{\partial v}{\partial t}+v\cdot \nabla v\right)=-\nabla p+\mu \nabla^{2}v+\rho g$$

where ρ is density, *p* is pressure, *g* is acceleration, and μ is a parameter. The Navier-Stokes equations are accompanied by a set of thermodynamic equations that interrelate temperature, pressure, and humidity within the atmosphere:

(27) $$\frac{\partial \rho }{\partial t}+\nabla \cdot \left(\rho v\right)=0\;\left(Continuity\;equation\right)$$

(28) $$\frac{\partial T}{\partial t}+v\cdot \nabla T=\frac{q}{c_{p}}\;\left(\mathrm{Energy}\;\mathrm{equation}\right)$$

(29) $$\frac{Dp}{Dt}=-\rho c_{p}\nabla \cdot v\;\left(\mathrm{Pressure}\;\mathrm{equation}\right)$$

where *T* is the temperature, and $c_{p}$ is a pressure parameter. The resulting solution gives the atmospheric state in each model grid cell.

This is very demanding computationally, and the solution can only be given over a relatively coarse grid. A postprocessing stage (mainly statistical in nature, possibly involving ML) is, therefore, needed to obtain data on a finer grid.

Over the past decades, the ability of NWP models to predict a future atmospheric state has continuously improved. Contemporary global NWP models are not only able to predict the synoptic-scale weather pattern for several days, but they have increased in their ability to predict regional events such as, e.g., major precipitation events.

The increasing success of operational NWP models is due to improvements in all the steps involved in the NWP workflow and the new capabilities (e.g., satellite measurements) of the global Earth observation system. The NWP model improvements can, therefore, be related in part to resolution enhancement. The continuous refinement of grid spacing has also required reformulating the dynamical cores of the NWP models, where the discretized Navier-Stokes equations are solved. Simulating the atmosphere at the kilometer scale brings with it the demand for highly parallelizable algorithms for the dynamical core [154]. Simultaneously, remarkable progress has been achieved in designing discretization approaches that enable the grid scale dynamics to follow the conservation laws of energy, entropy, and mass. Such physical conservation laws may cause problems in a pure ML approach. An extensive overview of contemporary dynamical core architecture and spatio-temporal forecasting can be found in [155].

The general development of ML, spanning over much more than weather forecasting and, in particular, of deep learning (DL), has had a history of advances and setbacks; see, e.g., Ref. [156]. Nowadays, ML has seen several instances of success. Recent developments are due to vastly increased computer capabilities, not the least due to massive parallel processing. This is the case with, for example, TCNs and transformers, as reported in Section 4.1. This has carried with it the hope that an ML system for weather forecasting may be faster than the present NWP system, allowing for more efficient processing of very large data sets. Finally, large benchmark data sets have become available on the internet. As a result of this development, highly complex neural network systems with more than $10^{6}$ parameters have enabled a breakthrough in image recognition, speech recognition, gaming, and video analysis.

The weather and climate community is, of course, aware of the powerful advances in ML. However, according to Ref. [156], there have still been reservations against ML methods, possibly due to the black box nature of ML methods and also the lack of implementation of physical conservation laws, as mentioned above; see [157]. Moreover, Ref. [158] argued that traditional ML methods may not yet be fully suitable for Earth System data; they claim that new network topologies are needed that not only exploit local neighborhoods (even at different scales) but also long-range relationships, for example, significant relationships or links between weather phenomena at widely separated locations on Earth, such as the effects from the El Niño Southern Oscillation (ENSO)). It may be of interest that both TCNs and transformers represent potential tools for modeling long-range relationships. See [159] for an example of transformers being used in long-term forecasting.

For some time now, there have been a number of uses of ML methods, but these have mainly been within more isolated parts of the weather prediction problem, such as the preprocessing and postprocessing stages mentioned earlier. In the preprocessing stage, ML methods can, for instance, be used in the interpolation of data to a regular grid. The postprocessing techniques have been reviewed in several papers, such as [160,161].

One survey [160] discusses both the use of parametric statistical distributions and ML methods. Distribution-based postprocessing approaches specify a parametric model for a forecast distribution. The parameters of the forecast distributions are then linked to the predictors from the NWP system via regression equations. Flexible additive regression functions are suggested in [162]. Despite these powerful variants, the need to select a suitable parametric family to describe the distribution of a target variable remains a limitation for parametric postprocessing methods and often necessitates elaborate fine-tuning.

When it comes to ML methods, random forest has been used in a variety of postprocessing situations, see, e.g., Ref. [163]. One challenge is, again, the interpretability of ML approaches. However, in weather forecasting, attempts have also been made to break open the black box; see, e.g., Ref. [164], which reviews methods for deciding which predictors are most important for a model and for a particular forecast. A more recent survey is contained in [157]. The authors discuss applications of SHAP and LIME, as mentioned in Section 4.3, to meteorological ML routines. They indicate how these interpretative aids may have the potential to gain more insight into predictions, uncovering novel meteorological relationships captured by machine learning. Further, they point out challenges around achieving deeper mechanistic interpretations aligned with physical principles. Refs. [165,166] should also be mentioned.

In general, combining forecasts (also in postprocessing) has been considered. Ref. [167] does this in the context of an ensemble Kalman filter. Again, one challenge regarding blending forecasts is to maintain the realistic physical features of the NWP model. There is, though, a clear shift [160] from physical modeling approaches to data-driven approaches. This shift is perhaps particularly transparent for the recent method of transformers, as discussed in Section 4.1. In [168], a transformer-based neural network is used in the postprocessing phase in predicting near-surface temperature, and in [169], hierarchical transformers are used in the postprocessing of temperature and precipitation forecasts. Moreover, transformers are used in precipitation nowcasting in [170].

These examples are restricted in that they are not full weather forecasts. This is also the case with some TCN (again, see Section 4.1) applications. Ref. [171] used TCN and LSTM for weather forecasting using time series data from local weather stations. TCN is also an important component in [172] in a hybrid model for wind power forecasting.

Despite its publication in Annals of Applied Statistics, Ref. [161] represents a more theoretical approach to postprocessing. The authors stress that accurate modeling of multivariate dependencies is crucial in many practical applications in general and, particularly, in NWP. In several approaches to postprocessing, ensemble predictions are first postprocessed separately in each margin, and multivariate dependencies are next established via copulas. Ref. [161] proposes a multivariate postprocessing method based on generative machine learning as an output of a general neural network to avoid some of the limitations regarding the two-stage copula approach. This results in a new class of nonparametric data-driven distributional regression models.

While this kind of postprocessing still requires a core of NWP or is restricted to one or a few meteorological variables, simultaneously, intensive work has been going on to try to establish a scheme of weather forecasts that are based entirely on statistical and/or ML approaches. Very recently, there has been remarkable progress in this area, which may even be a breakthrough.

The group behind this breakthrough is the Google Deep Mind group. They have taken ideas from the mechanism of transformers and used them in a wider context. They are extending the framework to graphs, which seems to be a more fitting model environment for weather forecasts due to the observations (nodes) being scattered over space and with dependence not only between neighboring points but also over larger distances referring to long-range meteorological phenomena.

A gentle introduction to graph neural networks is given in [173]. The connection between transformers and graph neural networks, with the former as a special case of the latter, is described in a Graph Deep Learning Lab report [174].

The GraphCast module developed for weather forecasting by the Google Deep Mind group is described in a Science 2023 article [175]; see also [176]. GraphCast is able to make medium-range weather forecasts with unprecedented accuracy. According to the authors, it predicts weather conditions up to 10 days in advance more accurately and much faster (inherited from the fast transformer routine set-up) than the industry gold standard weather simulation system—High Resolution Forecast (HRES) produced by the European Centre for Medium Range Weather Forecasts. GraphCast can also offer earlier warnings of extreme weather events.

GraphCast makes forecasts at the high resolution of 0.25 degrees longitude/latitude (28 km × 28 km at the equator). That is more than a million grid points covering the entire surface of the Earth. At each gridpoint, the model predicts five Earth-surface variables—including temperature, wind speed and direction, and mean sea level pressure—and six atmospheric variables. GraphCast is autoregressive; it can be “rolled out” by feeding its own predictions back in as input to generate an arbitrarily long trajectory of weather states.

The model was trained using 39 years (1979–2017) of historical data from existing archives. A training objective was to average the mean squared errors between GraphCast’s predicted states over a set of autoregressive steps and the corresponding data found in the archives. Much more details can be found in the Science paper.

What makes the Google Deep Mind group especially impressive is that the group has come up with another module that seems to be on the level of GraphCast, if not better. It is described in a late 2024 Nature paper [177]. It uses the appellation GenCast and is based on conditional probabilities, a form of conditional independence, using diffusion modeling. It has the same resolution as GraphCast and roughly the same training period; it beats the ENS model, the ensemble forecast of the European Centre for Medium Range Forecasting, in speed and accuracy in 97.2% of 1320 targets.

Do these advances mean that numerical weather prediction models can be discarded in favor of machine learning methods? This is not a viewpoint held by scientists in this area. In [178], GraphCast is critizised for its possible lack of physical consistency. Furthermore, explainability, in spite of recent advances, is still an issue that needs further attention. Rare events are hard to model due to the difficulty of having limited data during the training period.

Can machine learning methods be extended to cover really long-range periods and even give clues to possible climate modeling? This question is raised among others in a Csiro report [179]. It is pointed out that while weather prediction is a “big-data” problem, climate projections represent a “small-data” problem, with relatively little available data.

On the other hand, there seems to be every reason to continue the search for good machine learning methods in weather prediction, not the least as an extremely valuable (and fast) complement to numerical weather forecasting. Hybrid models of physical models and AI models may be of special interest. In a recent Nature paper, the authors of Ref. [180] discuss the possibility of coupling the physics of general circulation models with machine learning models to obtain a hybrid model that may be able to produce useful simulations of long-range weather or even climate processes. Similar arguments are used in [158].

Ref. [156] concludes that the research on pure ML methods in weather forecasting is still in its infancy; the specific properties of weather data require the development of new approaches beyond the classical concepts from computer vision, speech recognition, and other typical ML tasks. The author’s conclusion is that there may be potential for end-to-end (“pure”) ML weather forecast applications to produce equal or better quality forecasts for specific end-user demands, especially if these systems can exploit small-scale patterns in the observational data that are not resolved in the traditional NWP model chain. Whether ML and DL will evolve enough to replace most or all of the current NWP systems cannot be answered at this point. In any case, according to Ref. [158], one might argue for more interaction between physical and deep learning systems.

## Data Availability

Not applicable.

## References

[1] Delgado-Bonal A., Marchak A. (2019). Approximate entropy and sample entropy: A comprehensive tutorial. Entropy.

[2] Gupta R., Drzazga-Szczesniak E., Kais S., Szczesnial D. (2024). Entropy corrected geometric Brownian motion. Sci. Rep..

[3] Otneim H., Berentsen G., Tjøstheim D. (2022). Local lead-lag relationships and nonlinear Granger causality: An empirical analysis. Entropy.

[4] Tjøstheim D., Otneim H., Støve B. (2022). Statistical Modeling Using Local Gaussian Approximation.

[5] Tjøstheim D., Otneim H., Støve B. (2022). Statistical dependence: Beyond Pearson’s *ρ*. Stat. Sci..

[6] Makridakis S., Hibon M. (2000). The M3-competition: Results, conclusion and implications. Int. J. Forecast..

[7] Brown R. (1956). Exponential Smoothing for Predicting Demand.

[8] Holt C. (1957). Forecasting trends and seasonals by exponential smoothing averages. Off. Nav. Res. Memo..

[9] Billah B., King M., Snyder R., Koehler A. (2006). Exponential smoothing model selection for forecasting. Int. J. Forecast..

[10] Pfeffermann D., Allon J. (1989). Multivariate exponential smoothing: Method and practice. Int. J. Forecast..

[11] Box G., Jenkins G. (1970). Time Series Analysis. Forecasting and Control.

[12] Tsay R. (2017). Multivariate Time Series Analysis: With R and Financial Applications.

[13] Assimakopoulus V., Nikolopoulos K. (2000). The theta model: A decomposition approach to forecasting. Int. J. Forecast..

[14] Nikolopoulos K., Thomakos D. (2019). Forecasting with the Theta Method: Theory and Appliaction.

[15] Ahmed N., Atiya A., Gayar N.E., El-Shishiny H. (2010). An empirical comparison of machine learning models for time series forecasting. Econom. Rev..

[16] Hyndman R., Billah B. (2003). Unmasking the theta method. Int. J. Forecast..

[17] Fiorucci J., Pellegrini T., Louzada F., Petropolus F., Koehler A. (2010). Models for optimizing the theta method and their relationship to state space models. Int. J. Forecast..

[18] Spilotis E., Assimakopoulus V., Makridakis S. (2020). Generalizing the theta method for automatic forecasting. Eur. J. Oper. Res..

[19] Kyriazi F., Thomakos D., Guerard J. (2019). Adaptive learning forecasting with applications in forecasting agricultural prices. Int. J. Forecast..

[20] Harvey A. (1990). Forecasting Structural Time Series Models and the Kalman Filter.

[21] Durbin J., Koopman S. (2012). Time Series Analysis by State Space Methods.

[22] Casals J., Garcia-Hiernaux A., Jerez M., Sotoca S., Trindade A. (2016). State-Space Methods for Time Series Analysis: Theory, Applications and Software.

[23] Villagas M., Pedregal D. (2018). Supply chain decision support systems based on a novel hierarchical forecasting approach. Decis. Support Syst..

[24] Makridakis S., Spiliotis E., Assimakopoulos V. (2018). Statistical and machine learning forecasting methods: Concerns and ways forward. PLoS ONE.

[25] Tong H., Lim K. (1980). Threshold autoregression, limit cycles and cyclical data (with discussion). J. R. Stat. Soc. Ser. B.

[26] Chan K. (1993). Consistency and limiting distribution of the least squares estimator of a threshold autoregressive model. Ann. Stat..

[27] Tong H. (1990). Non-Linear Time Series. A Dynamical System Approach.

[28] Tsay R. (1998). Testing and modeling multivariate threshold models. J. Am. Stat. Assoc..

[29] Cai B., Gao J., Tjøstheim D. (2017). A new class of bivariate threshold cointegration models. J. Bus. Econ. Stat..

[30] Yang L., Bai H., Ren M. (2024). Threshold quantile regression neural network. Appl. Econ. Lett..

[31] Haggan V., Ozaki T. (1981). Modelling nonlinear random vibrations using an amplitude-dependent autoregressive time series model. Biometrika.

[32] Teräsvirta T. (1994). Specification, estimation and evaluation of smooth transition autoregressive models. J. Am. Stat. Assoc..

[33] van Dijk D., Teräsvirta T., Franses P. (2002). Smooth transition autoregressive models—A survey of recent developments. Econom. Rev..

[34] Dempster A., Laird N., Rubin D. (1977). Maximum likelihood from incomplete data via the EM algorithm. J. R. Stat. Soc. Ser. B.

[35] Hamilton J. (1990). Analysis of time series subject to changes in regimes. J. Econom..

[36] Guidolin M., Pedio M. (2018). Essential of Time Series for Financial Applications.

[37] Hastie T., Tibshirani R. (1990). Generalized Additive Models.

[38] Sperlich S., Tjøstheim D., Yang L. (2002). Nonparametric estimation and testing of interaction in additive models. Econom. Theory.

[39] Huang J., Horowitz J., Weai F. (2010). Variable selections in nonparametric additive models. Ann. Stat..

[40] Kolassa S. (2020). Why the best point forecasts depends on the error or accuracy measure. Int. J. Forecast..

[41] Box G., Cox D. (1964). An analysis of transformations. J. R. Stat. Soc. Ser. B.

[42] Schmidhuber J. (2015). Deep learning in neural networks: An overview. Neural Netw..

[43] Hornik K., Stinchcombe M., White H. (1989). Multilayer feedforward network are universal approximators. Neural Netw..

[44] Tjøstheim D., Jullum M., Løland A. (2023). Statistical embedding: Beyond principal components. Stat. Sci..

[45] Mikolov T., Chen K., Corrado G., Dean J. (2013). Efficient estimation of word representations in vector space. arXiv.

[46] Mikolov T., Sutskever I., Chen K., Corrado G., Dean J. Distributed representation of words and phrases and their composability. Proceedings of the Advances in Neural Information Processing Systems 26: Proceedings Annual 27th Conference on Neural Information Processing Systems.

[47] Lim B., Zohren S. (2021). Time series forecasting with deep learning: A survey. Philos. Trans. R. Soc. A.

[48] Gu J., Wang Z., Kuen J., Ma L., Shahroudy A., Shuai B., Liu T., Wang X., Wang G., Cai J. (2018). Recent advances in convolution neural networks. Pattern Recognit..

[49] Lea C., Vidal R., Reiter A., Hager G. Temporal convolution networks: A unified approach to action segmentation. Proceedings of the Computer Vision-ECCV 2016.

[50] Wan R., Mei S., Wang J., Liu M., Yang F. (2019). Multivariate temporal convolution network: A deep neural networks approach for multivariate time series forecasting. Electronics.

[51] Gopali S., Abri F., Namini S., Namin A. A comparison of TCN and LSTM models in detecting anomalies in time series data. Proceedings of the 2021 IEEE International Conference on Big Data.

[52] Salinas D., Bohlke-Schneider M., Callot L., Medico R., Gasthaus J. (2019). High-dimensional multivariate forecasting with low rank Gaussian copula processes. Advances in Neural Information Processing Systems.

[53] Rangupuram S., Seeger M., Gasthaus J., Stella L., Wang Y., Januschowski T. (2018). Deep state space models for time series forecasting. Advances in Neural Information Processing Systems.

[54] Lim B., Zohren S., Roberts S. Learning independent Bayesian filtering steps for time series prediction. Proceedings of the International Joint Conference on Neural Networks.

[55] Hochreiter S., Schmidhuber J. (1997). Long short-term memory. Neural Comput..

[56] Vaswani A., Shazeer N., Parmar N., Urzkoreit J., Jones L., Gomez A., Kaiser L., Polosukhin I. (2017). Attention is all you need. arXiv.

[57] Han K., Xiao A., Wu E., Guo J., Xu C., Wang Y. (2021). Transformer in transformer. arXiv.

[58] Su L., Zuo X., Li R., Wang X., Zhao H., Huang B. (2025). A systematic review for transformer-based long-term series forecasting. Artif. Intell. Rev..

[59] Grigsby J., Wang Z., Nguyen N., Qi Y. (2021). Long-range transformers for dynamic spatiotemporal forecasting. arXiv.

[60] Hastie T., Tibshirani R., Friedman J. (2019). The Elements of Statistical Learning.

[61] Freund Y., Schapire F. (1997). A decision-theoretic generalization of on line learning and an application to boosting. J. Comput. Syst. Sci..

[62] Ke G., Meng Q., Finley T., Wang T., Chen W., Ma W., Ye Q., Liu T.-Y., Gyon I., Luxburg U., Bengio S., Wallach H., Fergus K., Vishwanathan S., Garnett R. (2017). Light-GBM A highly efficient gradient boosting tree. Advances in Neural Information Processing Systems.

[63] Wang M., Hamidian N., Wisam E. What Are the Best Practices for Scaling Machine Learning Algorithms with Big Data. Posted on Linkedin April 2024. https://www.linkedin.com/advice/0/what-best-practices-scaling-machine-learning-algorithms-nxh1c.

[64] Palifka B. Understanding Machine Learning Algorithms: Training Time and Inference Time Complexity. Posted on Linkedin August 2024. https://www.linkedin.com/pulse/understanding-machine-learning-algorithms-training-time-bill-palifka-sqike.

[65] Ying X. (2018). An overview of overfitting and its solutions. J. Phys. Conf. Ser..

[66] Hassija V., Chamola V., Mahapatra A., Singai A., Goel D., Huang K., Scardapane S., Spinelli I., Mahmud M., Hussain A. (2024). Interpreting black-box models: A review on explainable artificial intelligence. Cogn. Comput..

[67] Azodi C., Tang J., Shiu S.H. (2020). Opening the black box: Interpretable machine learning for genetists. Trends Genet..

[68] Medeiros M., Vasconcelos G., Veiga Á., Zilberman E. (2021). Forecasting inflation in a data-rich environment: The benefits of machine learning methods. J. Bus. Econ. Stat..

[69] Shapley L. (1951). Notes on the n-Person Game-II: The Value of an n-Pearson Game.

[70] Lundberg S., Lee S.I. A unified approach to interpreting model predictions. Proceedings of the NIPS’17: Proceedings of the 31st International Conference on Neural Information Processing Systems.

[71] Lundberg S., Erion G., Lee S.I. (2018). Consistent individualized feature attribution for tree ensembles. arXiv.

[72] Ribeiro M., Singh S., Guestrin C. Why should I trust you?: Explaining the predictions of any classifier. Proceedings of the ACM SIGKDD International Conference on Knowledge Discovry and Data Mining.

[73] Visami G., Bagli E., Chesani F., Poluzzi A., Capuzzo D. (2020). Statistical stability indices for LIME obtaining reliable explanations for machine learning models. arXiv.

[74] Nohara Y., Matsumoto K., Soejima H., Nakashima N. (2022). Explanation of machine learning models using Shapley additive explanation and application for real data in hospital. Comput. Methods Programs Biomed..

[75] Rudin C. (2019). Stop explaining black box machine learning models for high stakes decision and use interpretable models instead. Nat. Mach. Intell..

[76] Hyndman R. (2020). A brief history of forecasting competitions. Int. J. Forecast..

[77] Makridakis S., Andersen A., Carbone R., Fildes R., Hibon M., Lewandowski R., Newton J., Parzen E., Winkler R. (1982). The accuracy of extrapolation (time series) methods: Results of a forecasting competition. J. Forecast..

[78] Makridakis S., Chatfield C., Hibon M., Lawrance M., Mills T., Ord K., Simmons L. (1993). The M2 competition: A real-time judgementally based forecasting study. Int. J. Forecast..

[79] Makridakis S., Spiliotis E., Assimakopoulos V. (2020). The M4 competition: 100,000 time series and 61 forecasting methods. Int. J. Forecast..

[80] Makridakis S., Spiliotis E., Assimakopoulos V. (2022). M5 accuracy competition: Results, finding and conclusions. Int. J. Forecast..

[81] Makridakis S., Spiliotis E., Holly R., Petropoulos F., Swanson N., Gaba A. (2023). The M6 forecasting competition: Bridging the gap between forecasting and investment decisions. arXiv.

[82] Small S. (2020). A hybrid method of exponnetial smoothing and recurrent neural metworks in time series forecasting. Int. J. Forecast..

[83] Bates J., Granger C. (1969). The combination of Forecasts. J. Oper. Res..

[84] Claeskens G., Magnus J., Vasnev A., Wang W. (2016). The forecast combination puzzle: A simple theoretical explanation. Int. J. Forecast..

[85] Taleb N. (2010). Black Swan—The Impact of the Highly Improbable.

[86] Fry C., Brundage M. (2020). The M3 forecasting competition—A practioneer’s view. J. Forecast..

[87] Farrel M., Liang T., Misra S. (2021). Deep neural networks for estimation and inference. Econometrica.

[88] Otneim H., Tjøstheim D. (2018). Conditional density estimation using local Gaussian correlation. Stat. Comput..

[89] Diebold F., Mariano R. (1995). Comparing predictive accuracy. J. Bus. Econ. Stat..

[90] Gneiting T., Ranjan R. (2011). Comparing density forecasts using threshold -and quantile -scoring rules. J. Bus. Econ. Stat..

[91] Gneiting T. (2008). Editorial: Probabilistic forecasting. J. R. Stat. Soc. Ser. A.

[92] Gneiting T., Katzfuss M. (2014). Probabilistic forecasting. Annu. Rev. Stat. Its Appl..

[93] Lerch S., Thorarinsdottir T., Ravazzolo F., Gneiting T. (2017). Forecaster’s dilemma: Extreme events and forecast evaluation. Stat. Sci..

[94] Gneiting T., Ranjan R. (2013). Combining predictive distributions. Electron. J. Stat..

[95] Salinas D., Flunkert V., Gasthaus J., Januschowsi T. (2020). DeepAR: Probabilistic forecasting with autoregressive recurrent networks. Stat. Sci..

[96] Croston J. (1972). Forecasting and stock control for intermittent demands. Oper. Res. Q..

[97] Gasthaus J., Benidis K., Wang Y., Rangapuram S., Salinas D., Flunkert V., Januschowski T. Probabilistic forecasting with spline quantile function RNNs. Proceedings of the 22nd International Conference on Artificial Intelligence and Statistics AIS-TATS.

[98] Wen R., Torkkola K. (2019). Deep generative quantile-copula models for probabilistic forecasting. ICML Time Series Workshop. arXiv.

[99] Engle R. (1982). Autoregressive conditional heteroscedasticity with estimates of the variance of United Kingdom inflation. Econometrica.

[100] Engle R., Bollerslev T. (1986). Modelling the persistence of conditional variances. Econom. Rev..

[101] Shephard N., Andersen T., Andersen T., Davis R., Kreiss J.P., Mikosch T. (2009). Stochastic volatility: Origins and overview. Handbook of Financial Time Series.

[102] Andersen T., Bollerslev T., Diebold F., Ebens H. (2001). The distribution of realized stock return volatility. J. Financ. Econ..

[103] Barndorff-Nielsen O., Shephard N. (2002). Econometric analysis of realized volatility and its use in estimating stochastic volatility models. J. R. Stat. Soc. Ser. B.

[104] Liu L., Patton A., Sheppard K. (2015). Does anything beat 5-minute RV: A comparison of realized measures across multiple asset classes. J. Econom..

[105] Zhang C., Zhang Y., Cucuringu M., Qian Z. (2024). Volatility forecasting with machine learning and intraday commonality. Financ. Econom..

[106] Andersen T., Bollerslev T., Meddahl N. (2004). Analytical evaluation of volatility forecasts. Int. Econ. Rev..

[107] Izzeldin M., Hassan M., Pappas V., Tsionas M. (2019). Forecasting realised volatility using ARFIMA and HAR models. Quant. Financ..

[108] Karolyi G., Lee K.H., Dijk M.V. (2012). Understanding commonality in liquidity around the world. J. Financ. Econ..

[109] Dang T., Moshirian F., Zhang B. (2015). Commonality in news around the world. J. Financ. Econ..

[110] Corsi F. (2009). A simple approximate long-memory model of realized volatility. J. Financ. Econ..

[111] Amirshahi B., Lahmiri S. (2023). Hybrid deep learning and GARCH-family models for forecasting volatility of cryptocurrencies. Mach. Learn. Appl..

[112] Almog A., Shmuli E. (2019). Structural entropy: Monitoring correlation-based networks over time with applications to financial markets. Sci. Rep..

[113] Wang S., Khan S., Munir M., Alhajj R., Khan Y. (2022). Entropy-based financial asset pricing: Evidence from Pakistan. PLoS ONE.

[114] Musmeci N., Aste T., Matteo T. (2016). Interplay between past market correlation structure changes and volatility outbursts. Sci. Rep..

[115] Rodriguez-Rodriguez N., Miramontes O. (2022). Shannon entropy: An econophysical approach to Cryptocurrency portfolios. Entropy.

[116] Drzazga-Szczesniak E., Szczpanik P., Kaczmarek A. (2023). Entropy of financial time series due to the shock of war. Entropy.

[117] Shih S.Y., Sun F.K., Lee H.Y. (2019). Temporal pattern attention for multivarite time series forecasting. Mach. Learn..

[118] Nketiah E., Chenlong L., Yingchuan J., Aram S. (2023). Recurrent neural network modeling of multivariate time series and its application to temperature forecasting. PLoS ONE.

[119] Kumar P., Priyanka P., Dhanya J., Uday K., Dutt V. (2023). Analyzing the performance of univariate and multivariate machine learning models in soil movement prediction: A comparison study. IEEE Access.

[120] Pesaran M., Pick A., Timmerman A. (2024). Forecasting with panel data: Estimation uncertainty versus parameter heterogeneity. arXiv.

[121] Chronopoulos I., Chrysikou K., Kapetanios G., Mitchell J., Raftapostolos A. (2023). Deep neural network estimation in panel models. arXiv.

[122] Engle R., Granger C. (1987). Cointegration and error correction: Representation, estimation and testing. Econometrica.

[123] Johansen S. (1988). Statistical analysis of cointegrating vectors. J. Econ. Dyn. Control.

[124] Escribano A., Wang D. (2021). Mixed random forest, cointegration, and forecasting gasoline prices. Int. J. Forecast..

[125] Du J. (2022). Mean-variance portfolio optimization with deep learning based-forecasts for cointegrated stocks. Expert Syst. Appl..

[126] El-Menshawry M., Ellwa M., EL-Essa L., El-Morshedy M., Sagheer R. (2024). Enhancing integer time series model estimation through neural-based fuzzy time series analysis. Symmetry.

[127] Snyder R., Ord J., Beaumont A. (2012). Forecasting the intermittent demand for slow-moving inventories. Int. J. Forecast..

[128] Rydberg T., Shephard N. (2000). BIN models for trade-trade data. Modelling the number of trades in a fixed time interval. Econometric Society World Congress 2000 Contributed Papers.

[129] Ferland R., Latour A., Oraichi D. (2006). Integer-valued GARCH process. J. Time Ser. Anal..

[130] Fokianos K., Rahbek A., Tjøstheim D. (2009). Poisson autoregression. J. Am. Stat. Assoc..

[131] Weiss C. (2018). An Introduction to Discrete-Valued Time Series.

[132] Eyjolfsson H., Tjøstheim D. (2018). Self-exiting jump processes with applications to energy markets. Ann. Inst. Stat. Math..

[133] Eyjolfsson H., Tjøstheim D. (2023). Multivariate self-exciting jump processes with applications to financial data. Bernoulli.

[134] Aït-Sahalia Y., Cacho-Diaz J., Laeven R. (2015). Modeling financial contagion using mutually exciting jump processes. J. Financ. Econ..

[135] Malaviya J. (2020). Survey on modeling intensity function of Hawkes process using neural models. arXiv.

[136] Creal D., Koopman S., Lucas A. (2013). Generalized autoregressive score models with applications. J. Appl. Econom..

[137] Artemova M., Blasques F., von Brummelen J., Koopman S. (2022). Score-driven models: Methodology and theory. Oxford Research Encyclopedia of Economics and Finance.

[138] Newman M. (2020). Networks.

[139] Crane H. (2018). Probabilistic Foundations of Statistical Network Analysis.

[140] Tjøstheim D., Jullum M., Løland A. (2023). Some recent trends in embeddings of time series and dynamic networks. J. Time Ser. Anal..

[141] Zhu X., Pan R., Li G., Liu Y., Wang H. (2017). Network vector autoregression. Ann. Stat..

[142] Ludkin M., Eckley I., Neal P. (2018). Dynamic stochastic block models: Parameter estimation and detection in community structure. Stat. Comput..

[143] Krampe J. (2019). Time series modeling on dynamic networks. Electron. J. Stat..

[144] Armillotta M., Fokianos K., Krikidis I. Generalized linear models network autoregression. Proceedings of the International Conference on Network Science.

[145] Armillotta M., Fokianos K. (2021). Poisson network autoregression. arXiv.

[146] Armillotta M., Fokianos K. (2022). Testing linearity for network autoregressive models. arXiv.

[147] Armilotta M., Fokianos K. (2023). Nonlinear network autoregression. Ann. Stat..

[148] Richardson L. (1922). Weather Prediction by Numerical Process.

[149] Wiin-Nielsen A. (1991). The birth of numerical weather prediction. Tellus.

[150] Lynch P. (2008). The origens of computer weather prediction and climate modeling. J. Comput. Phys..

[151] Bauer P., Thorpe A., Brunet G. (2015). The quiet revolution of numerical weather forecasting. Nature.

[152] Bannister R. (2007). A review of operational methods of variational and ensemble variational data assimilation. Q. J. R. Meteorol. Soc..

[153] Chen L., Han B., Wang X., Zhao J., Yang W., Yang Z. (2023). Machine learning methods in weather and climate applications. Appl. Sci..

[154] Krasnopolsky V., Fox-Rabinovitz M., Hou Y., Lord S., Belochiski A. (2010). Accurate and fast neural network emulations of model radiation to the NCEP coupled climate forecast system: Climate simulations and seasonal predictions. Mon. Weather Rev..

[155] Markakis E., Papadopoulos A., Perakis P. (2018). Spatio-temporal forecasting: A survey. arXiv.

[156] Schultz M., Betancourt C., Gong B., Kleinert F., Langgut M., Leufen L., Mozaffari A., Stadtler S. (2020). Can deep learning beat numerical weather forecasting?. Philos. Trans. A.

[157] Yang R., Hu J., Li Z., Mu J., Yu T., Li J.X., Dasgupta A., Xiong H. (2024). Interpretable machine learning for weather and climate prediction: A review. Atmos. Environ..

[158] Reichstein M., Camps-Valls G., Stevens B., Jung M., Denzler J., Carvalhais J. (2019). Deep learning and process understanding of data-driven earth system science. Nature.

[159] Lee T., Roy S., Kumar A., Ramachandran R., Nair U. Long term forecasting of environment variables of MERRA2 based on transformers. Proceedings of the European Geosciences Union (EGU) General Assembly 2023.

[160] Vannitsen S., Bremnes J.B., Demaeyer J., Evans G., Flowerdew J., Hemri S., Lerch S., Roberts N., Theis S., Atencia A. (2021). Statistical postprocessing for weather forecasts: Review, challenges, and avenues in a big Data World. Bull. Am. Meteor. Soc..

[161] Chen J., Janke T., Steinke F., Lerch S. (2024). Generative machine learning methods for multivariate postprocessing. Ann. Appl. Stat..

[162] Righby R., Stasinopoulos D. (2005). Generalized additive models for location, scale and shape. J. R. Stat. Socitey Ser. C.

[163] McGovern A., Elmore K., Gagne D., Haupt S., Kartens C., Lagerquist R., Smith T., Williams J. (2017). Using artificial intelligence to improve real-time decision-making for high-impact weather. Bull. Am. Meteor. Soc..

[164] McGoverns A., Jergerson G., Elmore K., Homeyer C., Smith T. (2019). Understanding the physical implications of machine learning. Bull. Am. Meteor. Soc..

[165] Molnar C. *Interpretable Machine Learning. A Guide for Making Black Box Models Explainable*; Independently Published, 2022. https://christophm.github.io/interpretable-ml-book/.

[166] Miftachov R., Keilbar G., Härdle W. (2024). Shapley curves: A smoothing perspective. J. Bus. Econ. Stat..

[167] Nerini D., Foresti L., Leuenberger D., Robert S., German U. (2019). A reduced space ensemble Kalman filter approach for flow-dependent integration for radar extrapolation nowcasts and NWP precipitation ensembles. Mon. Weather Rev..

[168] Alerskans E., Nyborg J., Birk M., Kaas E. (2022). A transformer neural network for predicting near-surface temperature. Meteorol. Appl..

[169] Bouallegue Z., Weyn J., Clare M., Dramsch J., Dueben P., Chantry M. (2024). Improving medium-range ensemble weather forecasts with hierarchical ensemble transformers. Artif. Intell. Earth Syst..

[170] Piron M.J., Wang X., Kim H., Kwon H. (2024). Precipitation nowcasting using transformer-based generative models and transfer learning for improved disaster preparedness. Int. J. Appl. Earth Obs. Geoinf..

[171] Hewage P., Behera A., Trovati M., Pereira E., Ghahremani M., Palmieri F., Liu Y. (2020). Temporal convolution neural (TCN) network for an effective weather forecasting using time series data from the local weather station. Springer Nat. Link.

[172] Zhu J., Su L., Li Y. (2022). Wind power forecasting based on new hybrid model with TCN residual modification. Energy AI.

[173] Sanchez-Lengeling B., Reif E., Pearce A., Wiltschko A. (2021). A gentle introduction to graph neural networks. Distill.

[174] Joshi C. (2020). Transformers Are Graph Neural Networks.

[175] Lam R., Sanchez-Gonzales A., Willson M., Wirnsberger P., Battaglia P., Fortunato M., Alet F., Ravuri S., Ewalds T., Eaton-Rosen Z. (2023). Learning skillful medium-range global weather forecasting. Science.

[176] Keisler R. (2022). Forecasting global weather with graph neural networks. arXiv.

[177] Price I., Sanchez-Gonzales A., Alet F., Andersson T., El-Kadi A., Masters D., Ewalds T., Stott J., Mohamed S., Battaglia P. (2024). Probabilistic weather forecasting with machine learning. Nature.

[178] Bonavita M. (2024). On some limitaions of current machine learning weather prediction models. Geophys. Res. Lett..

[179] Kitsios V. (2024). AI Weather Models Can Now Beat the Best Traditional Forecasts.

[180] Kochkow D., Yuval J., Langmore I., Norgaard P., Smith J., Mooers G., Klöwer M., Lottes J., Rasp S., Düben P. (2024). Neural general circulation models for weather and climate. Nature.

